# From Label‐Free Multiphoton Imaging to Pathological Reports: A Vision‐Language Breast Cancer Margin Pathological Diagnosis System

**DOI:** 10.1002/advs.75709

**Published:** 2026-05-15

**Authors:** Shu Wang, Jingze Su, Xiahui Han, Deyong Kang, Xiao Zhang, Fei Xu, Changzu Liu, Junlin Pan, Xingfu Wang, Qiaohui Zhan, Aimin Wang, Feng Huang, Heping Cheng, Wenxi Liu, Ruolan Lin, Jianxin Chen

**Affiliations:** ^1^ School of Mechanical Engineering and Automation Fuzhou University Fuzhou China; ^2^ National Biomedical Imaging Center State Key Laboratory of Membrane Biology Institute of Molecular Medicine Peking‐Tsinghua Center for Life Sciences College of Future Technology Peking University Beijing China; ^3^ Fujian Provincial Key Laboratory of Photonics Technology Key Laboratory of OptoElectronic Science and Technology for Medicine of Ministry of Education Fujian Normal University Fuzhou China; ^4^ College of Computer and Data Science Fuzhou University Fuzhou China; ^5^ Department of Pathology Fujian Medical University Union Hospital Fuzhou China; ^6^ Department of Pathology The First Affiliated Hospital of Fujian Medical University Fuzhou China; ^7^ Department of Breast Surgery The Second Affiliated Hospital of Xiamen Medical College Xiamen China; ^8^ State Key Laboratory of Photonics and Communications School of Electronics Peking University Beijing China; ^9^ Department of Radiology Fujian Medical University Union Hospital Fuzhou China

**Keywords:** breast cancer, multiphoton microscopy, surgical margin, tumor‐associated collagen signatures, tumor microenvironment, virtual staining, vision‐language model

## Abstract

Margin pathological assessment provides critical feedback for breast‐conserving surgery, whereas H&E‐stained histopathology focuses on residual tumor and may cause unnecessary resections. Label‐free multiphoton microscopy (MPM) reveals tumor‐associated collagen signatures (TACS) at the margin, offering a prognostically relevant diagnostic information. However, limited familiarity of novel MPM images by pathologists has prevented its integration into diagnostic workflows. Here, we introduce MarginPath, a Margin Pathological diagnosis system built on an MPM‐language model that requires only a single label‐free section. By integrating MPM‐derived TACS with virtual H&E diagnostic information, MarginPath provides a multimodal diagnostic report, including: (i) a MPM image and corresponding virtual H&E image, (ii) a TACS‐based pixel‐level margin‐status heatmap, and (iii) detailed natural‐language diagnostic descriptions of tumor margin microenvironment. Validated on 158 invasive breast cancer specimens, MarginPath outperforms existing pathology vision‐language models in margin diagnosis and can be extended into a question‐answering system, enhancing both clinical decision‐support and patient communication.

## Graphical abstract

MarginPath is a novel vision‐language system that automates breast cancer margin assessment using a single label‐free multiphoton microscopy image. By integrating tumor‐associated collagen signatures with virtual H&E imaging, it generates accurate margin heatmaps and comprehensive diagnostic reports. This multimodal approach effectively bridges advanced optical imaging with clinical histopathology to enhance surgical decision‐making.

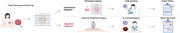

## 1 Introduction

Breast cancer is the most prevalent malignancy in women worldwide [1, 2]. Early‐stage disease is commonly treated with breast‐conserving surgery (BCS) [3], aiming to achieve adequate tumor excision while preserving maximal breast volume [4]. In practice, intraoperative H&E‐stained pathological diagnosis evaluates surgical margins by focusing on tumor detection [5, 6], yet it is often blind to prognostically relevant tumor microenvironment (TME) components, potentially causing imprecise resection due to false‐negative margins.

Multiphoton microscopy (MPM), a next‐generation label‐free histopathology validated across 16 human organs, can simultaneously capture both collagen fibers and tumor cells within the TME, offering a valuable complement to traditional histopathology [7, 8, 9]. In breast cancer, MPM has uncovered eight types of prognostically relevant tumor‐associated collagen signatures (TACS). Notably, TACS4 to TACS8, located at the tumor boundary or invasive front, play a critical role in facilitating local recurrence and metastasis [10, 11, 12, 13].

However, MPM faces two principal barriers to clinical adoption. First, the absence of formal training programs leaves pathologists unfamiliar with MPM image contrast, hindering the practical application of multiphoton‐derived TACS for margin assessment. Second, although deep‐learning models trained on MPM images can improve diagnostic accuracy [14] and prognostic prediction [15], the overall pathology workflow remains laborious. Consequently, practitioners require not only supplementary multiphoton TACS visualizations but also concise, decision‐level textual diagnosis that can be integrated seamlessly into existing workflows.

Recent advances in vision–language models (VLMs) have enabled automated generation of pathology descriptions from histological images, with proof‐of‐concept studies spanning multiple cancer types [16, 17]. In breast cancer, large language‐vision models (LLMs) have been applied mainly to molecular subtyping [18]; margin assessment remains largely unexplored. Although FastGlioma [19] deployed stimulated Raman scattering for rapid intraoperative detection of infiltrative glioma margins, the algorithm focused on tumor cells and neglected critical TME cues, limiting diagnostic robustness. Concurrent work has improved fine‐grained feature recognition by extending analysis from image patches [20] to whole‐slide images (WSI) [21]. Yet WSI‐level models are susceptible to hallucinated captions, undermining reliability in clinical workflows. Although spatial cross‐verification can partially mitigate this, current WSI‐based architectures still struggle to resolve complex tissue morphologies and TME components [22]. Consequently, there remains an unmet need for multimodal VLMs that exploit advanced imaging contrasts, such as MPM, to accurately characterize the intricate TACS during margin evaluation.

Here, we present MarginPath, a comprehensive system for pathological margin diagnosis built upon a novel MPM‐language model. Using a single label‐free tissue section, MarginPath streamlines the workflow from multiphoton imaging to diagnostic reporting. It generates a bimodal diagnostic signature by combining multiphoton‐based TACS with virtual H&E‐derived tumor cell information, making it accessible to pathologists regardless of their MPM expertise. The system simultaneously provides complementary multimodal outputs: (i) a virtual H&E image corresponding to the input MPM image, (ii) a margin status heatmap, and (iii) detailed diagnostic descriptions capturing comprehensive tumor margin characteristics. Validated on 158 invasive breast cancer specimens, MarginPath captures more comprehensive imaging and diagnostic information than conventional pathology reports, outperforming existing pathology VLMs in margin diagnosis. This capability significantly enhances decision‐making support for both pathologists and surgeons during margin assessment and prognostic evaluation.

## 2 Results

### 2.1 MarginPath Workflow

**FIGURE 1:**
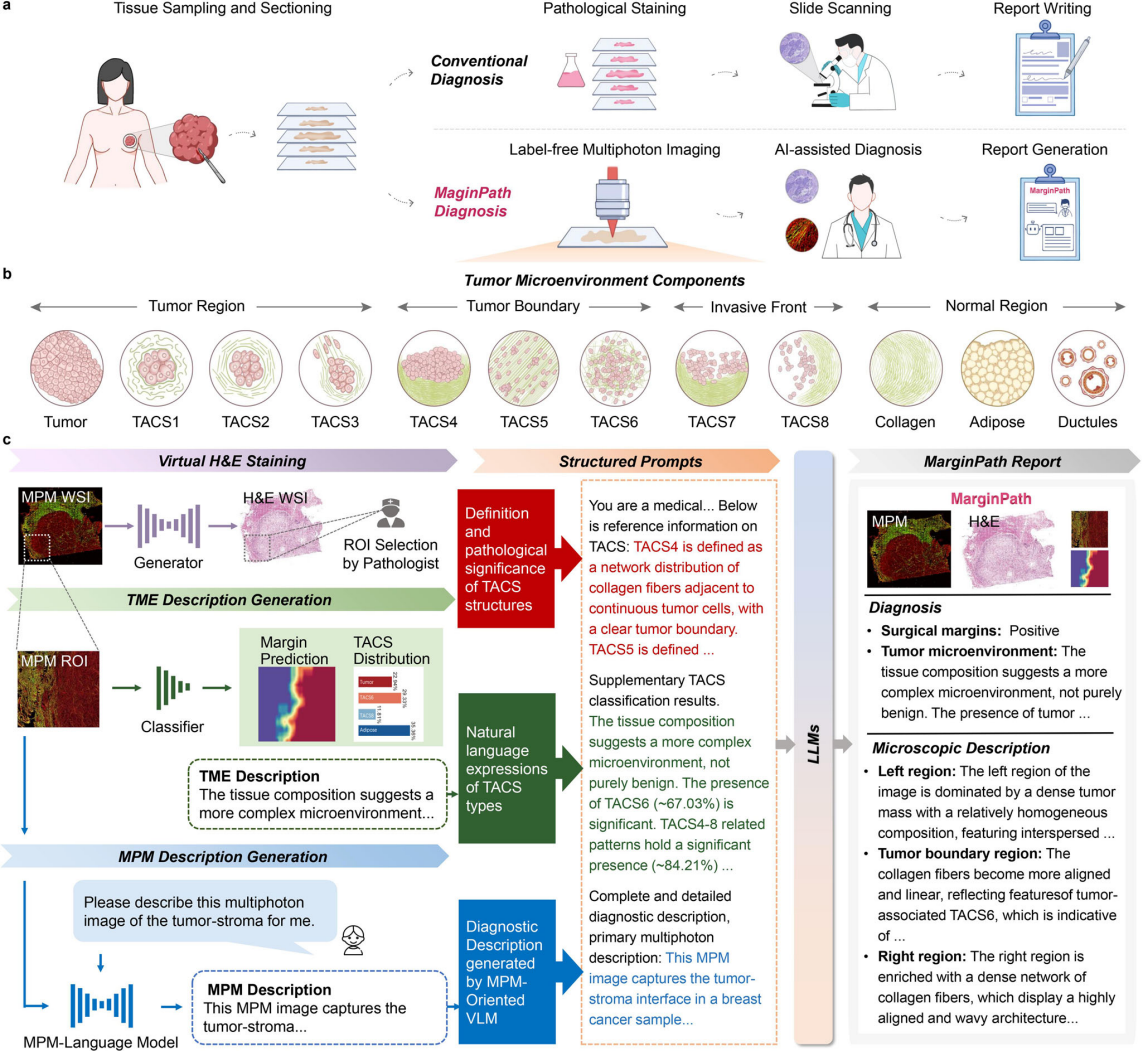
Workflow of our proposed pathological diagnosis system MarginPath. (a) and (b) Comparison with conventional pathology workflow, MarginPath enables label‐free, specific imaging of TME components, particularly TACS relevant to surgical margin status, using only a single unlabeled tissue section. TME features are visualized to establish a dual‐modal margin diagnostic signature combining MPM and H&E images. Simultaneously, it leverages MPM images and corresponding virtual H&E‐stained images to automatically produce a comprehensive pathology report. (c) Overview of the MarginPath diagnosis pipeline, which comprises the following steps. First, a label‐free WSI is acquired through MPM, and the WSI MPM image can be virtually stained into an H&E image. Given the virtually stained H&E image, the pathologist can screen out suspicious ROIs. Next, MarginPath performs classification diagnosis on the ROIs. The classification results and ROI images are converted into structured prompts to facilitate report generation. Finally, the comprehensive pathological report, including images and texts, is generated. The image part includes the WSI MPM image, the virtually stained WSI H&E image, the MPM image of the suspicious ROI, and the corresponding margin heatmap. The text part includes the margin positivity or negativity, the TME composition, and a complete description.

The proposed MarginPath diagnosis pipeline comprises the following steps (Figure 1). First, a label‐free WSI is acquired through MPM. Specifically, by leveraging second harmonic generation (SHG) and two‐photon excited fluorescence (TPEF), MPM captures key TME components at the margin, including tumor cells, TACS1 through TACS8, ductal lobular structures, and adipose tissues, by focusing on four critical regions: the tumor region, tumor boundary, invasive front, and adjacent normal region. Importantly, TACS patterns exhibit distinct spatial distributions: TACS1 to TACS3 are primarily located within the tumor region, TACS4 to TACS6 reside at the tumor boundary, and TACS7 and TACS8 are found at the invasive front. Consequently, TACS4 to TACS8, which reflect dynamic tumor‐stroma interactions at the periphery, provide more significant prognostic information and thereby enhance the dimensionality of margin diagnosis.

Second, the WSI MPM image is virtually stained into an H&E image from an unsupervised intensity‐inversion‐based virtual staining network. The motivation is twofold: (1) Combining MPM and H&E imaging, TME features can be clearly visualized to establish a dual‐modal margin diagnostic signature; and (2) the pathologist can easily screen out suspicious regions of interest (ROIs) according to the virtually stained H&E image that enhances MPM pathological interpretability. Then, the proposed MarginPath system performs classification diagnosis targeting TACS features based on a classifier. It can effectively predict categorical probability of TME components and visualize the tumor margin status to assist pathologists in identifying tumor boundary and invasive front within virtual H&E images, where discrimination is challenging.

Third, the ROI images and their corresponding classification diagnosis are converted into structured prompts to facilitate pathology report generation. To do so, we develop a novel MPM‐language model that enables holistic diagnostic description generation for MPM images. The generated MPM caption emphasizes diagnostically critical features, such as tumor‐stroma interfaces, by precisely describing tissue architectures and spatial relationships in MPM images. In addition, we parse the TME classification results into their natural language expressions along with the TACS definitions and pathological significance as structured prompts.

Finally, MarginPath can produce a comprehensive and illustrated pathological report including images and text. The image part includes the WSI MPM image, the virtually stained WSI H&E image, the MPM image highlighting the suspicious ROI, the corresponding probability map of tumor margin status, and TACS compositional charts. The textual component is produced by passing the structured prompts into an LLM, which provides the explanation of margin status, TME compositional analysis, and region‐specific microscopic description, delivering TME‐dimensional insights beyond conventional clinical pathology reports.

### 2.2 MPM‐H&E Bimodal Margin Diagnostic Signatures

**FIGURE 2:**
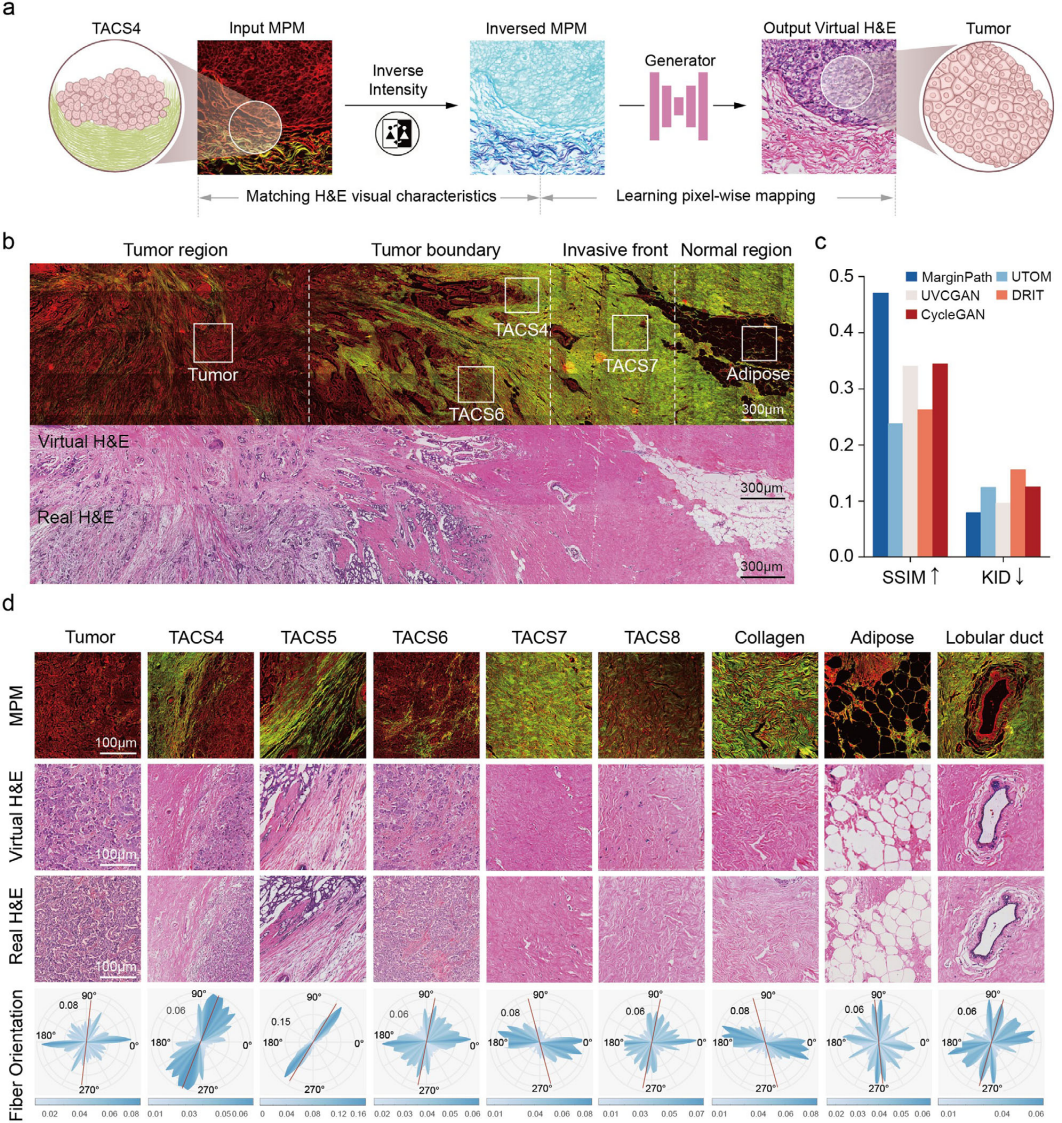
MarginPath reveals TME features of MPM images and the corresponding virtual H&E‐stained images. (a) The virtual staining network performs intensity inversion on MPM images to generate corresponding virtual H&E images, establishing a precise mapping for collagen and tumor structures within TACS across both modalities. (b) Representative MPM and virtual H&E‐stained images of tumor‐stroma interface in invasive breast cancer, showing the locations of TACS. (c) MarginPath outperforms the competing virtual staining methods for the MPM‐to‐H&E image transformation task in terms of SSIM and KID metrics, indicating higher similarity between generated and reference fiber images (*n *= 17,636 patches). (d) Nine representative TME features along with their corresponding virtual/real H&E images, and collagen fiber orientation distributions (red line: mean orientation of fibers, color bar: probability density of fibers). These elements define an MPM‐H&E bimodal diagnostic signature by integrating MPM‐derived TACS4 to TACS8 (located at the tumor boundary and invasive front) with virtual H&E‐derived tumor cell information, enabling a comprehensive assessment of the TME at the surgical margin.

Pathologists typically assess margin status by identifying tumor cells. However, collagen remodelling at tumor boundaries also reflects invasive potential. To address limitations in conventional histopathological diagnosis, Figure 2 illustrates the proposed novel diagnostic approach based on multiphoton TACS alongside corresponding virtual H&E images.

Due to the significant differences in visual style and intensity features between MPM images and H&E‐stained images, in contrast to H&E images, pathologists lack an intuitive understanding and recognition ability for MPM images. Thus, to enhance the interpretability of MPM imaging in breast cancer margin diagnosis, we first develop an unsupervised virtual staining model (Figure 2a). Conventional virtual staining networks tend to produce erroneous transformations for collagen fibers and cells, due to the stark contrast between the H&E bright background and the MPM dark background, as well as the absence of pixel‐level supervision.

To overcome this concern, we introduce a simple yet effective pixel intensity inversion strategy. Specifically, real MPM images are inverted before being processed by an unsupervised CycleGAN [23] to generate virtual H&E images. This process establishes a more accurate pixel‐level mapping between collagen and cellular structures in H&E and MPM modalities, thereby substantially improving the accuracy of image conversion and its diagnostic utility. Figure 2b displays representative MPM and virtual H&E images from an invasive breast carcinoma. Virtual H&E images provide superior cellular morphological clarity compared to MPM, with higher SSIM and lower KID metrics than existing methods (Figure 2c and Figure S1). For the location of TACS (Figure 2b), the leftmost panel depicts the tumor region, rich in tumor cells with scant collagen, corresponding to the primary location of TACS1 to TACS3 [10, 24]. The tumor boundary region displays TACS4 to TACS6 collagen morphologies‐hallmarks of advanced invasion stages associated with prognosis‐that arise from TME remodeling [12, 25]. In the rightmost panel, TACS7 and TACS8 are typically located at the invasive front, distal to the tumor boundary within histologically normal regions.

Regarding the histopathological characteristics of TACS, Figure 2d provides enlarged illustrations of nine typical TME features from the tumor region, tumor boundary, invasive front, and normal region, including lobular duct, collagen fibers, adipose tissue, TACS4 to TACS8, and tumor cells (related TPEF and SHG images are provided in Figure S2). Specifically, TACS4 to TACS6 contain variable numbers of tumor cells, often aligned along more focused collagen fibers. TACS4 features a reticular collagen network adjacent to an expanding tumor mass with well‐defined borders, while TACS6 displays disorganized collagen fibers, indistinct boundaries, and multidirectional tumor cell migration along collagen strands. In contrast, TACS7 and TACS8 exhibit distinct collagen fiber densities, with a distribution as random as that of normal collagen. Lobular duct and adipose tissue show sparse and randomly oriented collagen. Quantitative comparison of TACS4 to TACS8 is provided in Figure S3.

Based on these observations, we establish that the MPM‐H&E bimodal margin diagnostic signature delineates key prognostic regions by mapping the tumor‐stroma boundary and the invasive front as a critical transition zone, based on distinct TACS patterns. This paradigm enhances margin assessment accuracy, thereby providing a scientific basis for personalized postoperative therapy. Notably, TACS1 to TACS3 are excluded from the bimodal signature as they are predominantly located within the tumor core and are less relevant for evaluating the surgical margin.

### 2.3 Margin Visualization Based on Multiphoton Tumor‐Associated Collagen Signatures

**FIGURE 3:**
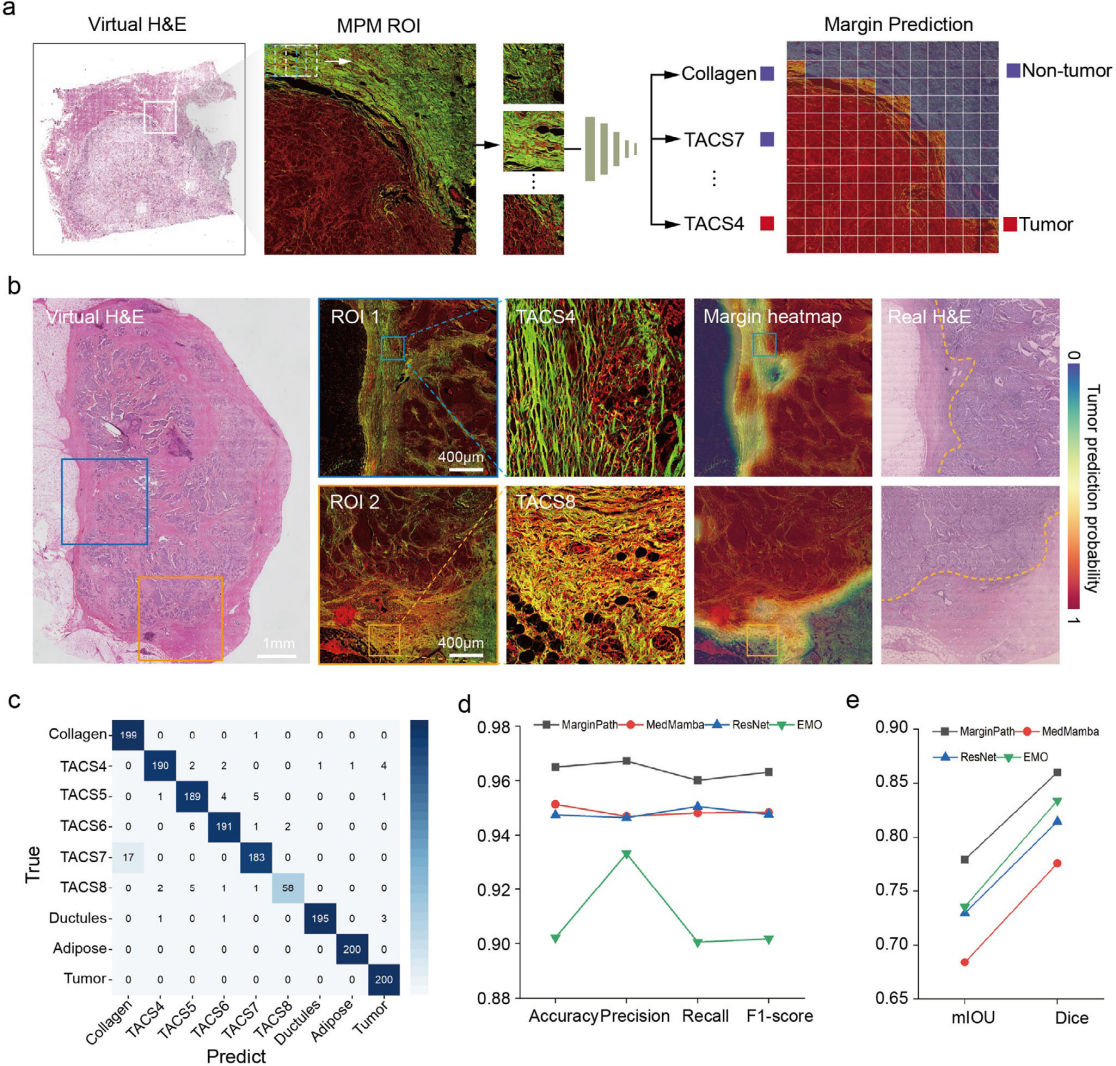
Tumor margin visualization using MarginPath. (a) Illustration of the tumor margin visualization process based on multiphoton TACS. (b) A representative case of invasive breast cancer showing the visualization results using MPM images. The blue and orange boxes highlight the ROIs selected by pathologists from virtual H&E images. The corresponding margin heatmap visualizes the tumor and non‐tumor regions based on tumor prediction probability. Regions with high probability are classified as tumor regions and displayed in red; those with moderate probability, defined as suspected regions, are shown in yellow; and regions with low probability, corresponding to non‐tumor regions, are rendered in blue‐purple. The margin position is consistent with the ground truth (the orange dashed line in real H&E images). (c,d) MarginPath shows superior performance in discriminating TME feature categories, outperforming other methods in TME classification (*n *= 1667 patches) and margin prediction (*n *= 66 ROIs).

Leveraging our established MPM‐H&E bimodal diagnostic signatures, we develop a breast cancer margin visualization pipeline (Figure 3a) based on a Vision Transformer (ViT) [26] classification model. The pipeline comprises three steps: ROI screening, TME classification, and margin prediction. Specifically, pathologists first identify suspicious ROIs within H&E‐stained WSIs. Subsequently, the classification model analyzes each image patch according to a set of TME features, including TACS4 to TACS8 structures, peritumoral adipose tissue, ductules, and tumor tissue. The model assigns each patch to a diagnostic category: “Tumor” (containing tumor cells and TACS4 to TACS6) and “non‐tumor” (encompassing TACS7 and TACS8 in the absence of tumor cells, as well as adjacent normal tissue). Finally, these patch‐level classifications are integrated. The prediction probability of each class is color‐coded and superimposed onto the corresponding MPM image to generate a comprehensive margin assessment heatmap (the details are provided in the Methods Section on tumor margin visualization).

Figure 3b demonstrates an example of invasive breast carcinoma. Particularly, in ROI‐1, the collagen‐rich stromal regions with adipose transition into tumor tissue. In contrast, ROI‐2 displays densely packed tumor cells superiorly and collagen inferiorly, with an indistinct boundary. The tumor‐stroma interface exhibits dense, interwoven bundles, while adjacent collagen appears fragmented and disorganized, reflecting matrix degradation and remodeling. Then, MarginPath generates tumor margin heatmaps within ROIs, displaying high‐probability tumor regions (red) that align with H&E ground truth, low‐probability non‐tumor regions (blue‐purple), and crucial moderate‐probability regions (yellow) at the tumor periphery, suggesting the presence of ambiguous yet critical TME features. For example (Figure 2b), TACS4 in ROI‐1 manifests as a dense, parallel collagen band along the tumor boundary, with upward extensions showing looser fibers interwoven with infiltrating tumor cells, a hallmark of tumor‐stroma interaction. In contrast, TACS8 in ROI‐2 presents sparsely distributed collagen fibers at the tumor invasion front, largely devoid of tumor cells. This enhanced visualization proves particularly valuable for assessing subtle or poorly defined margins, as demonstrated in Figure S3a.

Figure 3c validates MarginPath's superior performance in discriminating TME feature categories. Notably, the occasional misclassification of TACS7 as normal stroma further validates that the yellow, moderate‐probability peri‐tumoral regions harbor ambiguous, prognostically relevant TME features. Moreover, MarginPath outperforms MedMamba [27], ResNet [28], and EMO [29] architectures across the accuracy, precision, recall, and F1‐score metrics, demonstrating enhanced discriminative power and generalizability for TME features. Notably, MedMamba and ResNet show comparable overall accuracy but exhibited weaker discrimination between structurally similar classes (e.g., TACS5 vs. TACS6; see Figure S4b). For margin prediction, MarginPath achieves the highest mIoU and Dice scores, confirming its superior ability to precisely delineate tumor margins (Figure 3d).

### 2.4 Transformation of Margin Images Into Textual Descriptors

**FIGURE 4:**
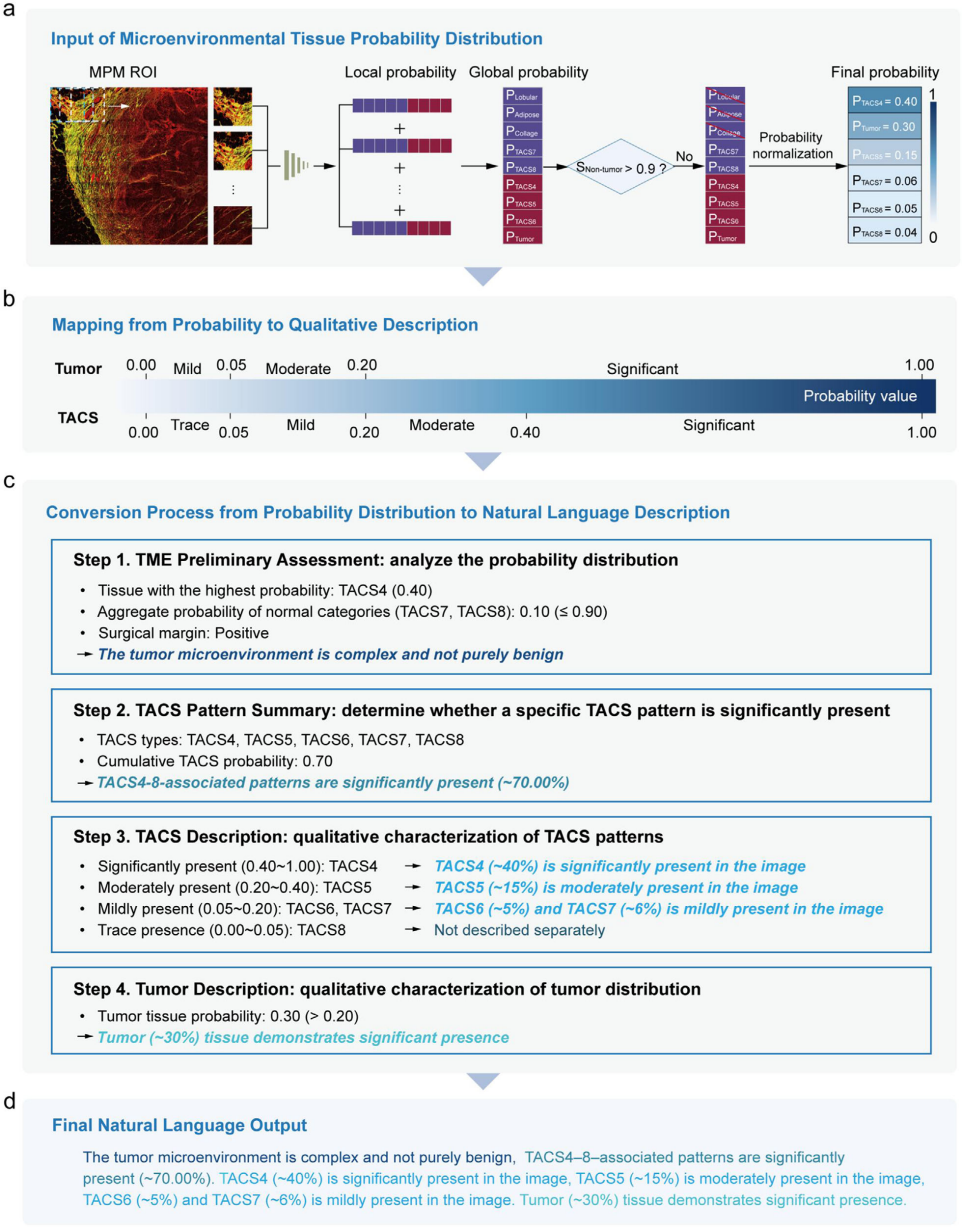
Converting diagnostic class probability prediction of an MPM ROI into natural language as structured prompts. (a) Overview of the process from MPM image input to global class probability prediction. (b) Mapping the tumor and TACS probabilities to natural language descriptions. (c) An example illustrating the conversion pipeline via the four steps: (1) preliminary TME assessment; (2) TACS pattern summarization; (3) TACS description; and (4) tumor description. (d) Final natural language output generated from the probability distribution as structured prompts.

Translating TACS classification probability of an ROI sample into interpretable textual descriptions is crucial for generating interpretability pathology reports. As shown in Figure 4a, we develop a clinical significance level description reasoning algorithm. This algorithm first aggregates tumor classification probabilities of patches extracted from the ROI as the holistic class probability through weighted averaging and normalization. Next, the probabilities of classification as normal tissue and TACS are quantified, and their clinical significance is assigned according to predefined probability ranges. As shown in Figure 4b, we convert continuous diagnostic probabilities into discrete levels of clinical significance, including “Significant,” “Moderate,” “Mild,” and “Trace.” Please refer to Section 4.5 for the detailed implementation of the proposed algorithm.

Taking Figure 4c as an example, the procedure comprises four steps: (1) In the TME assessment step, MarginPath determines overall microenvironment status based on the combined probability of non‐tumor tissue categories, such as normal collagen, adipose, ductules, TACS7, TACS8. ROIs are classified as “Predominantly non‐tumor tissue features” where probabilities exceed 0.9; otherwise, “The tumor microenvironment is complex and not purely benign” is assigned. (2) In TACS pattern summary step, MarginPath highlights the most prevalent tissue type, prioritizing TACS4 to TACS8 and tumor tissue. (3) In TACS description step, MarginPath provides tiered descriptions of each TACS pattern's prominence, aiding assessment of tumor infiltration dynamics. (4) In the tumor description step, MarginPath reports the probability of tumor cell tissue, further providing evidence for the margin status.

This algorithm enables high‐fidelity mapping from probability distributions to structured text. By providing semantically rich prompts, particularly capturing the expressiveness of MPM‐derived TACS patterns and tumor boundary features, it supplies accurate contextual information for MarginPath. This enhances both the professionalism and semantic consistency of generated reports, thereby improving the clinical utility of MarginPath.

### 2.5 Multiphoton Margin Pathological Report Generation

**FIGURE 5:**
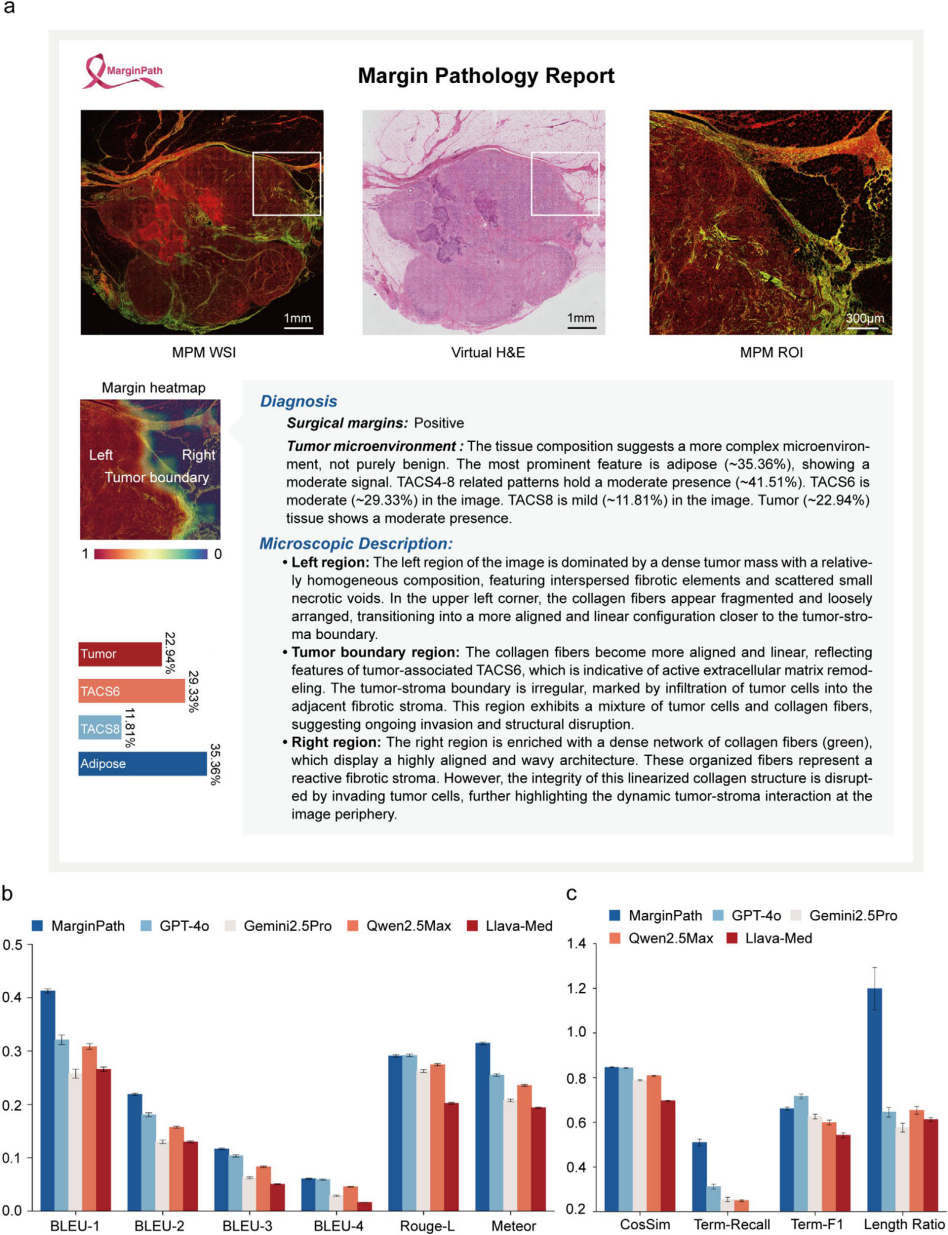
Multiphoton margin pathology report generation using MarginPath. (a) Example of a generated pathological report. This pathological report consists of two sections, incorporating bimodal images, diagnostic findings, and the description of TME. On the upper part, there are three figures, i.e., the MPM WSI, virtual H&E WSI, and MPM ROI, while the bottom part contains the tumor margin heatmap, diagnostic results, and microscopic descriptions. (b and c) MarginPath outperforms other vision‐language models in generating linguistically structured microscopic descriptions and medical applicability (*n* = 172 ROIs).

Figure 5a exemplifies a novel margin pathology report generated by MarginPath for a case of triple‐negative breast cancer, comprising the following components: MPM and virtual H&E images, margin heatmaps, and textual description. Virtual H&E WSI facilitates diagnostic interpretation of the MPM images. For regions of uncertainty in the H&E WSIs, ROIs are selected from the MPM image to generate localized margin heatmaps and corresponding textual descriptions.

The generated textual description is divided into a diagnostic result and a microscopic description. MarginPath not only produces a margin status concordant with the ground‐truth pathology report but also provides a comprehensive TME compositional analysis, thereby delivering a more information‐rich diagnostic readout. Specifically, the report indicates that the ROI demonstrates a “complex tumor microenvironment, nonbenign,” comprising 22.94% tumor tissue and 41.51% TACS4 to TACS8 components. To strengthen the readability, the tumor probability heatmap and the most likely classification for each ROI sample are visualized alongside the diagnostic conclusion. The corresponding textual descriptions for these elements are provided in the microscopic description section. In the left high‐probability region, predominantly dense tumor tissue with fragmented, loosely arranged collagen fibers. In the transition moderate‐probability region, an irregular tumor‐stroma interface with intermixed tumor cells and collagen, suggestive of active invasion and structural disruption. In the right low‐probability region, dense collagen network and adipose tissue, while fibers exhibit high alignment and waviness, with linear arrangements partially disrupted by invading tumor cells, highlighting dynamic tumor‐stroma interactions at the margin. A pathological report for another representative luminal B breast cancer case is presented in Figure S5.

To comprehensively evaluate report quality on the external test set of 26 patients, we assess its precision across two dimensions: linguistic similarity and medical applicability. For linguistic similarity (Figure 5b and Table S1), we use BLEU‐1 to BLEU‐4, ROUGE‐L, and Meteor to evaluate the accuracy at the language structure and vocabulary levels. MarginPath outperforms most comparison models, demonstrating a stronger ability to capture MPM image details and generate naturalistic diagnostic descriptions. Notably, its BLEU‐4 score is 5‐fold higher than that of LLaVA‐Med [30], indicating substantially improved performance in producing coherent and logically structured long‐form diagnostic text. For medical applicability (Figure 5c and Table S2), we further evaluate the models using Cosine Similarity (CosSim), Term‐Recall, Term‐F1, and Length Ratio. MarginPath achieves the highest semantic similarity and substantially outperforms the other methods in Term‐Recall, indicating a stronger ability to capture and express clinically relevant findings. Although GPT‐4o achieves slightly higher scores on ROUGE‐L and Term‐F1, its lower term recall and shorter generated reports suggest a more conservative reporting style with reduced clinical content coverage. In contrast, MarginPath achieves a near‐reference Length Ratio and broader coverage of diagnostically relevant features, supporting its ability to generate more comprehensive pathology reports while preserving domain‐specific semantic meaning.

Furthermore, we perform a detailed comparative analysis of the descriptions generated for the critical tumor‐stroma boundary in MPM images across different methods. As shown in Figure S6, MarginPath provides more structurally locational descriptions and more accurate TACS information. Finally, we conducted a comprehensive subjective evaluation of MarginPath's pathological reports, covering virtual staining quality, margin heatmap identification, and textual description accuracy (Note S1). The results indicate that while the virtual staining exhibits minor stylistic differences from real H&E, it remains fully adequate for diagnostic observation. Both the margin localization and the descriptive accuracy were rated as effective for guiding clinical decision‐making.

Additionally, MarginPath can be extended into a question‐answering system that incorporates a medical dialogue module to enhance clinician‐patient communication. As detailed in Figure S7 and Note S2, we developed a breast cancer question‐answering (Q&A) reasoning dataset from medical licensing examinations and implemented a group relative policy optimization (GRPO)‐based reinforcement learning strategy with a multi‐metric reward function. This significantly improved the dialogue model's medical reasoning capabilities. Figures S8 and S9 present example Q&A interactions with MarginPath from patients and clinicians, respectively.

To further assess the attention‐guiding value of MarginPath in diagnostically challenging peri‐margin regions, we perform a focused observer analysis on suspicious ROIs identified within the yellow pseudo‐colored regions of the heatmap from the independent test set (*n *= 26 patients). As shown in Note S3 and Figure S10, using expert consensus on the matched H&E‐stained sections as the reference standard, these highlighted ROIs achieve an accuracy of 92.31%, a sensitivity of 91.67%, and a specificity of 92.86%. These findings support the potential utility of the heatmap for localizing subtle tumor‐positive regions in challenging peri‐margin areas.

## 3 Discussion and Conclusions

Precise pathological diagnosis provides crucial feedback on margin status, thereby enabling more accurate excision. However, the identification of an adequate margin remains controversial in BCS [5, 31, 32]. Novel techniques have recently been deployed to resolve this issue. Combining preoperative and intraoperative ultrasound guidance may reduce tumor‐involved margin rates [33], but ultrasound sensitivity is limited by spatial resolution, constraining its utility for margin assessment. Optical coherence tomography (OCT) [34], an optical counterpart to ultrasound, leverages low‐coherence light interference to probe scattering properties of margin components. While OCT achieves superior spatial resolution and sensitivity, it is still requiring histological references for evaluation. Apart from the above techniques, MPM is increasingly used to refine tumor‐margin diagnosis because it can directly visualize the TME [35, 36]. Accumulating evidence shows that the TME actively drives tumorigenesis, progression, invasion, and metastasis [37, 38, 39], yet these features were largely ignored by earlier imaging strategies. TACS‐key structural elements of the TME‐have been detected both at the invasive margin and in distant stroma [25]. Recent MPM advances have elucidated the macro‐ and micro‐scale TACS features and their spatial relationships with invasive breast cancer prognosis [10, 11, 12]. Thus, incorporating TACS into margin pathology could provide valuable insights into cancer cell growth and escape, enabling more precise assessments.

Furthermore, the TACS‐based diagnostic paradigm proposed here may be extendable to other solid tumors. In addition to breast cancer, MPM has been applied to label‐free imaging of pancreatic [40], colorectal [41], and gastric cancers [42]. Moreover, although TACS was initially described in breast cancer, similar collagen‐remodeling patterns have increasingly been reported in other solid malignancies and are associated with invasion, metastasis, recurrence, and prognosis. These observations suggest that collagen‐based microenvironmental signatures may represent a more generalizable stromal biomarker framework across tumor types. Accordingly, the integrated architecture of MarginPath, combining MPM‐derived stromal feature characterization with structured vision‐language report generation, may provide a useful foundation for future adaptation to margin assessment and microenvironment‐aware diagnosis in other solid tumors.

In this work, we establish a more comprehensive diagnostic framework by integrating TACS‐derived stromal information from MPM with tumor‐cell information from H&E histology for tumor margin evaluation. While high‐resolution MPM provides complementary diagnostic information, it also increases interpretive complexity and the risk of information overload. To address this, we developed MarginPath, a diagnostic pipeline that integrates virtual staining, margin visualization, and pathology reporting based on bimodal diagnostic signatures. Virtual H&E staining facilitates interpretation of MPM images, patch‐wise TACS visualization enables assessment of diagnostically ambiguous regions, and the LLM‐driven report generator produces structured pathological descriptions for interactive review. Notably, MarginPath is designed to quantify TACS patterns rather than provide direct prognostic prediction, as prognosis depends on broader clinical and demographic factors.

Though large pathology models perform well in general domains, clinical decision‐making requires integration of pathological findings with broader patient information. MarginPath addresses this need by combining lightweight models for multiphoton feature extraction and semantic interpretation with large models for natural‐language report generation. To reduce hallucination and information loss, the system translates margin‐related statistical metrics and visual semantics into standardized textual descriptions and embeds TACS definitions and their pathological significance into structured prompts, thereby improving alignment with pathology reporting conventions and preserving interpretability.

For clinical translation, MarginPath is intended to complement the existing H&E workflow rather than replace routine histopathology. Label‐free MPM imaging can be performed before conventional staining, and the resulting virtual H&E images, heatmaps, and structured reports may help pathologists localize suspicious regions and support intraoperative assessment more efficiently. Because the same tissue section can subsequently undergo standard H&E staining, compatibility with current pathological procedures is preserved. Beyond diagnostic accuracy, turnaround time is an important factor for intraoperative use. The current MarginPath workflow requires approximately 11–16 min, compared with about 30 min for conventional frozen section examination, suggesting that the system may fit within the intraoperative workflow and help reduce surgical waiting time. By converting raw multiphoton information into more familiar visual and textual outputs, MarginPath may also reduce the learning burden associated with direct MPM interpretation.

Nevertheless, our approach has the following limitations. First, the performance of MarginPath is contingent on the quality and diversity of the training data, so it may decrease for rare specimens. Second, despite the integration of caption descriptions and structured prompts, LLMs may still generate factually inconsistent content, particularly under conditions of probabilistic ambiguity (e.g., co‐occurrence of TACS5 and TACS6), introducing hallucination risks in report outputs. As the training data diversity and data amount grow, the generalizability of MarginPath will be improved by decoding latent dataset patterns. In practical terms, it will offer immediate clinical benefits: surgeons gain more confident intraoperative decision support, enabling them to determine margin clearance with reduced uncertainty.

## 4 Methods

### 4.1 Ethical Statement

All anonymous tissue collections for retrospective study of MPM imaging were conducted under a protocol approved by the Clinical Research Ethics Committee of Fujian Medical University [2024KJT004] and Harbin Medical University Cancer Hospital [KY2020‐11].

### 4.2 Patients and Sample Preparation

Specimens were obtained from 158 breast cancer patients, aged 21 to 87 years, who had undergone tumor resection surgery. Inclusion criteria were as follows: (1) patients with pathologically confirmed invasive breast cancer, with no evidence of distant metastasis, who had received surgical treatment; and (2) patients with complete clinicopathological data and follow‐up records. Written informed consent was provided by all patients, or by authorized representatives where necessary, for the collection of tissue samples.

For sample preparation, tissue blocks containing the invasive front—defined as the boundary between the primary tumor and adjacent normal tissue—were sectioned using a cryostat. Two consecutive 5 µm‐thick sections were prepared from each block. Both sections underwent dewaxing with xylene and dehydration through a graded ethanol series. One section was reserved for MPM imaging without staining, while the other was stained with H&E. MPM images were acquired using a commercial confocal microscope (Zeiss LSM 880 META, Jena, Germany) coupled with a mode‐locked Ti: sapphire laser (140 fs, 80 MHz). The excitation wavelength and average power were set at 810 nm and 30 mW, respectively [43]. H&E‐stained slides were scanned using a commercial whole‐slide scanner (VM1000, Motic, China). A senior pathologist reviewed the H&E‐stained images to confirm the presence of tumor cells, TACS, and delineate tumor boundaries, which served as a reference for optical imaging analysis. Two optical specialists subsequently identified ROIs within the MPM images, focusing on TACS, which were annotated and co‐registered with the corresponding H&E images for further analysis.

### 4.3 MarginPath Datasets

Real‐world MPM images from 158 patients were assembled into a dedicated dataset. Images from 132 patients (Fujian Medical University Union Hospital) were used to train the three core modules of MarginPath; images from the remaining 26 patients (Harbin Medical University Cancer Hospital) were reserved for independent system‐level validation. The 132‐patient subset was further partitioned into three task‐specific training cohorts.

#### 4.3.1 MPM‐to‐H&E Virtual Staining Dataset

This sub‐dataset consists of 24 070 image patches from the MPM and H&E domains, extracted from whole‐slide images using a 512 × 512 sliding window with a stride of 256 (Table S3). For the virtual staining task, the dataset was strictly split at the patient level into independent training and testing sets containing 6434 and 17 636 patches, respectively, with no patient overlap between the two sets. Notably, the H&E whole‐slide images were acquired from adjacent tissue sections and were not pixel‐wise registered to the MPM images; therefore, the virtual staining model was trained under an unpaired image translation setting.

#### 4.3.2 Tumor Margin Visualization Dataset

A total of 16 654 image patches were extracted from WSIs using a 512 × 512 sliding window with a stride of 512 (Table S4). This sub‐dataset supports the training and evaluation of the TME classifier and enables the generation of pixel‐level tumor confidence maps. The data were divided into training, validation, and test sets in a ratio of 7:2:1. Each patch was annotated by senior pathologists into one of nine TME categories, including tumor, collagen, fat, lobular tissue, and TACS4 to TACS8, providing reliable ground truth for the ViT‐based classifier.

To independently assess the trained classifier's performance in tumor margin visualization, we introduced an additional dedicated test set comprising 66 MPM ROI images (average resolution: 4358 × 4358 pixels) that were not used during model training. All surgical margins in these ROIs were manually delineated by experts, offering gold‐standard annotations for evaluating visualization accuracy.

#### 4.3.3 MPM Description Generation Dataset

This sub‐dataset contains 276 MPM‐text pairs derived from 132 patients (average resolution: 4296 × 4322 pixels) and is used to train the MPM‐language model, enabling automated diagnostic description generation from MPM images. The external test set contains 172 MPM ROI images from the remaining 26 patients, reserved for final system evaluation (Table S5). Each image is paired with a diagnostic description (approximately 110–300 words) authored by senior pathologists. The text was refined with assistance from GPT‐4o [44] and includes spatial descriptors indicating the coordinates of collagen and tumor regions to improve localization accuracy and clinical interpretability.

### 4.4 MPM‐to‐H&E Virtual Staining

Before MPM can be used in clinical practice to assist in diagnosis, systematic training is usually required for pathologists, resulting in additional human and time costs. To effectively overcome the above challenges and further enhance the interpretability of MPM images, we developed an unsupervised deep learning model: an intensity‐inversion‐based virtual pathological staining generative adversarial network. This model transforms MPM images into virtual H&E images that are familiar to clinicians.

Specifically, we employed an unsupervised GAN‐based model to transform MPM images into virtual H&E images, thereby eliminating the need for pixel‐level registration between MPM and H&E‐stained images. In practice, images typically exhibit a dark background, whereas H&E images display a light background. This stark contrast in visual styles can easily cause the model to learn incorrect mappings during training, leading to significant deviations in the virtual staining results. Due to the lack of pixel‐level supervision, the classical GAN‐based model (e.g., CycleGAN [23]) tends to confuse the background with the tissue structures (Figure S1). To address this issue, we improved the classic GAN‐based architecture that incorporates an image intensity inversion mechanism (as shown in Figure S11). The intensity inversion mechanism reverses the pixel values of the MPM images, effectively reducing the stylistic differences between the two types of images and helping the network more easily learn the correlations between image features. Formally, given an input image *I_MPM_*, we first generate its intensity‐inverted version *I_reverse_* =  255 −  *I_MPM_*. This inverted image is then fed into the H&E generator $G_{H\&E}$ to produce the virtual H&E image. To optimize the network training process, we employed a combination of multiple loss functions for supervising this model, including adversarial loss [45], cycle‐consistency loss [23], identity loss [23], and structural similarity (SSIM) loss [46]. The overall loss is defined as:

(1) $$L=\lambda_{adv}L_{adv}+\lambda_{cycle}L_{cycle}+\lambda_{identity}L_{identity}+\lambda_{SSIM}L_{SSIM},$$

where the adversarial loss ensures the realism of the generated pathological staining images; the cycle‐consistency loss guarantees stability and reversibility between the multiphoton and pathological image domains; the identity loss preserves color and style consistency in the generated images; while the SSIM loss ensures that the generated virtual H&E images exhibit high local structural consistency with real stained images.

### 4.5 Tumor Margin Visualization

To enable precise identification of tumor boundaries in high‐resolution MPM ROI images, we defined a human‐in‐the‐loop workflow for ROI selection (Figure S12). In the current workflow, ROI selection is performed by an experienced pathologist based on the virtual H&E image. Specifically, when diagnostically ambiguous regions are identified, such as areas with scattered atypical cells, indistinct tumor boundaries, or complex desmoplastic stroma, the pathologist manually delineates a bounding box to define the ROI. The coordinates of the selected ROI are then recorded and mapped to the corresponding raw MPM whole‐slide image. Because the virtual H&E and MPM modalities are pixel‐wise registered, the matched high‐resolution MPM ROI can be automatically extracted from the recorded coordinates. Subsequent TME classification and tumor margin visualization are performed on the extracted MPM ROI, yielding a diagnostic probability heatmap for boundary assessment.

In the TME classification stage, we adopted a ViT as the foundational model (i.e., ViT‐Base‐Patch16‐224 [26]) and conducted supervised training on the tumor margin visualization dataset to achieve a TME classifier that enables accurate recognition of distinct TME regions. Specifically, under the standard ViT pipeline, each input MPM image is spatially decomposed into a sequence of 16 × 16‐pixel patches, each of which is linearly projected into 768‐dimensional token embeddings. Combined with learnable positional encodings that preserve spatial information, the resulting sequence of token embeddings is subsequently processed by a 12‐layer Transformer encoder [47] and gains the image feature representation, where each layer incorporates multi‐head self‐attention modules and a feed‐forward network with residual connections and layer normalization. Finally, based on the feature representation, the TME classifier can estimate the probability distribution over the target categories.

Next, in the margin‐prediction stage, an overlapping sliding‐window strategy was deployed to apply the patch‐level prediction from the trained classifier, yielding a pixel‐wise tumor‐confidence map that precisely localizes the tumor boundary. Specifically, for each MPM ROI, a sliding window with a stride of 128 pixels is applied to partition it into a series of overlapping patches of size 512 × 512 pixels. Each patch is independently fed into a ViT classifier to obtain its corresponding tissue category prediction. To further visualize the tumor margin, all predicted categories are aggregated into two classes: “non‐tumor” and “tumor.” Based on the sliding‐window strategy, each pixel is covered by 16 overlapping patches. For each pixel location, we count the number of times it is classified as “tumor” and divide that count by 16 to obtain the predicted probability of that pixel belonging to tumor. In this way, a unified tumor probability map is generated at the original image resolution, where the value of each pixel (ranging from 0 to 1) reflects the likelihood that the location corresponds to tumor‐associated tissues. For better visualization, the resulting confidence map is rendered using a spectral color scheme, thereby clearly revealing the spatial distribution of the tumor and its infiltrative boundary.

### 4.6 TME Description Generation

Directly employing LLMs to parse high‐resolution MPM images for generating diagnostic descriptions poses a fundamental challenge, as these models struggle to extract diagnostically meaningful global semantic features directly from pixels [48]. To bridge the semantic gap between the ROI sample and clinical reports, we develop a hierarchical probabilistic fusion and diagnostic reasoning framework. The core of this framework involves a stepwise aggregation of the patch‐level probability predicted by the tumor margin classifier into a global diagnostic conclusion for the target ROI sample, which is ultimately converted into a qualitative natural language description, thereby transforming visual information into structured high‐level concepts understandable by LLMs.

#### 4.6.1 ROI Tumor Diagnostic Probability

This step aims to integrate dispersed local information into a holistic understanding of the sampled ROI. Specifically, we evenly crop the target ROI sample into a set of smaller patches and then compute the classification probability for each patch $p_{i}\left(p_{i}\in R^{1\times 9}\right)$ through the tumor margin classifier. Last, we employ a confidence‐weighted averaging strategy to combine the local classification probabilities as the holistic probability, as below:

(2) $$P=\sum_{i}c_{i}\;\cdot \;p_{i},$$

where *c_i_* represents the confidence of the probability prediction for the i‐th patch and *P*(*P* $\in R^{1\times 9})$ refers to the holistic diagnostic probability. Instead of directly estimating the probability for the ROI sample, this local‐to‐global strategy involves the confidence of categorizing local patches into particular tissue types (i.e., normal collagen, adipose tissue, ductal‐lobular structures, TACS4‐8, and tumor).

#### 4.6.2 From Classification Probability to Clinical Captions

To improve interpretability in clinical reporting, MarginPath maps continuous diagnostic probabilities to discrete significance levels (“Trace,” “Mild,” “Moderate,” and “Significant”). This design was adopted for two reasons. First, categorical descriptors are more intuitive than raw probabilities for rapid pathological assessment. Second, they support more stable and standardized language generation, improving the readability and consistency of the generated diagnostic text. Based on this strategy, the final description includes a global TME assessment, an overall TACS pattern summary, specific TACS descriptions, and tumor‐related findings.

To simplify the process, we divide the tissue types into two sets according to the multiphoton and H&E diagnostic signatures: a non‐tumor tissue set *S*_*non* − *tumor*_ and a tumor‐associated tissue set *S_tumor_*, as shown in Table 1. Thus, we can measure the probability of the ROI sample being classified as non‐tumor, denoted as *p*_*non* − *tumor*_, by aggregating the probabilities of being classified as the categories in *S*_*non* − *tumor*_, as below:

(3) $$p_{non\;-\;tumour}=\;\sum_{i\in S_{non\;-\;tumour}}P[i],$$

where *P*[*i*] refers to the *i*‐th item of the probability vector *P*.

**TABLE 1:** Categorization of tissue types into non‐tumor and tumor‐associated sets.

Symbol	Tissue categories
*S*_*non* − *tumor*_	{Normal collagen, Adipose tissue, Ductal‐lobular structures, TACS7, TACS8}
*S_tumor_*	{TACS4, TACS5, TACS6, Tumor}

When *p*_*non* − *tumor*_ is larger than 0.9, we can produce the global TME assessment as the text prompt “*the tumor microenvironment is predominantly normal*.” Under these circumstances, we set the item of *P* belonging to *S_tumor_*, i.e., *P*[*i*]$\left(i\in S_{tumor}\right)$, as zeros and re‐normalize the estimated class probability *c*. Next, to summarize the TACS patterns, we only consider the probability of being classified as TACS7 and TACS8, i.e., *P*[*TACS7*] and *P*[*TACS8*]. According to Table S6, we can map the probability to a discrete clinical significance level. Concretely, we can produce the TACS overall pattern‐related text prompt “*TACS7‐8‐associated patterns are [CSL] presented [∼PROB]*,” where *[CSL]* represents the clinical significance level according to the sum of *P*[*TACS7*] and *P*[*TACS8*]. [PROB] refers to the value of the corresponding probability. Additionally, we can produce the text descriptions for TACS7 and TACS8, respectively. For example, the text prompt of TACS7 can be phrased as “*TACS7 [∼PROB] is [CSL] present in the image*.”

On the other hand, when *p*_*non* − *tumor*_ is smaller than 0.9, we let the global TME assessment be “*a complex tumor microenvironment, not purely benign*” and we set the items of *P* belonging to *S*_*non* − *tumor*_, i.e., $P\left[i\right]\left(i\in S_{non-tumor}\right)$, as zeros and re‐normalize the estimated class probability *P*. Next, we measure the probability of being classified as TACS4‐8 to create the TACS overall pattern prompts. Similarly, we can create the respective text descriptions for TACS4‐8. In addition, we also produce the text prompt for the probability of tumor class. By contrast to TACS, the correspondence of the probability and significance level for the tumor differs, as depicted in Table S7. Accordingly, we can easily produce the tumor description as “*Tumor [∼PROB] tissue presents [CSL]*.”

### 4.7 MPM Description Generation

Existing foundation models are restricted to H&E‐stained histology; to our knowledge, none generate descriptions for label‐free MPM images. To develop an image captioning model specifically for MPM images, we adopt the pretrained VLM (i.e., the Qwen2‐VL‐2B model [49]) as our base architecture. This model is selected due to its strong performance in image understanding and language generation in medical image captioning tasks. Its key advantage lies in supporting dynamic resolution input, which enables effective handling of the large dimensions and complex histopathological features of MPM images, while avoiding information loss caused by cropping. Furthermore, its robust multilingual semantic comprehension and deployment flexibility make it well‐suited for developing automated image analysis and diagnostic assistance tools in clinical environments.

The VLM architecture integrates a vision encoder and an LLM to jointly process visual and semantic information from MPM images and generate diagnostic text (Figure S13). The ViT‐based vision encoder converts input images into visual tokens; its support for dynamic resolution input combined with 2D rotary position encoding (2D‐RoPE) effectively enhances the model's perception of visual information across different scales. To improve efficiency, the visual tokens are first compressed spatially via a multilayer perceptron that merges adjacent tokens in a 2 × 2 pattern. They are then projected to match the LLM's feature dimensions through a vision adapter. These tokens are subsequently fed into a cross‐modal fusion module equipped with multimodal RoPE, which enhances localization in structurally complex regions (e.g., tumor boundaries) by modeling positional relationships. Finally, a self‐attention mechanism jointly models the image and text tokens to ensure semantic alignment. On the other hand, the LLM decoder generates text autoregressively. It is optimized for processing long sequences and high‐resolution image information, incorporating a hierarchical attention mechanism to dynamically focus on diagnostically relevant regions. During inference, token compression is applied to reduce redundant computations, accelerating generation while preserving textual accuracy and coherence.

Based on the architecture described above, we conduct specialized training of the model. During the training phase, we fine‐tune it for three epochs on our custom MPM description dataset using the AdamW optimizer [50] with a learning rate of 1 × 10^−^^5^ and a cosine annealing schedule [51]. To adapt the model to the new task while preserving the powerful visual representations it originally possesses, we freeze the parameters of the vision encoder and update only the weights of the remaining components. The training objective is to minimize the cross‐entropy loss between the text generated by the model and the reports annotated by pathologists. The dataset examples are illustrated in Figure S14.

After fine‐tuning, this VLM demonstrates excellent capabilities in pathological image understanding and report generation during the inference phase. The descriptions generated for MPM images accurately capture the core pathological structure of the “tumor stroma interface” and provide reasonable explanations of the spatial distribution of tumor tissue and collagen regions. Particularly notable is the high degree of consistency between the model's descriptions of “collagen fiber organization” and the ground truth pathological reports. Furthermore, the model effectively identifies transitional changes in collagen density within the images. These performances indicate that our constructed model possesses good generalization ability in cross‐modal information alignment and in interpreting the complex spatial structures of MPM images, validating the potential of our method for clinical applications.

### 4.8 Pathological Report Generation

In the final stage, we deliver the above‐generated structured prompts to an off‐the‐shelf LLM to obtain the textual report. As shown in Figure 1, the structured prompts comprise of three parts. (1) Definition and pathological significance of typical TACS structures, e.g., “*TACS4 is defined as a network distribution of collagen fibers adjacent to continuous tumor cells …*”, which enables pathologists to advance their understanding of TACS significance; (2) TME description that describes the ROI condition and tumor margin status in natural language; and (3) MPM description generated by our fine‐tuned VLM. Finally, these parts are composited together as the input to the LLM. In this way, the generated report follows three core principles:

#### 4.8.1 Semantic Hierarchy and Role Clarity

MPM description provides the primary semantic representation of macroscopic MPM features. In contrast, TACS classification results act as supplementary inputs, used to insert missing microenvironmental features and correct inconsistencies, thereby enhancing logical coherence and reducing redundancy.

#### 4.8.2 Embedding Medical Knowledge

To enhance the LLM's domain‐specific understanding in multiphoton pathology, prompts incorporate predefined descriptions of typical TACS structures and their pathological significance (e.g., “*TACS4 corresponds to early‐stage collagen remodeling at the tumor boundary*”), guiding the LLM to interpret TACS roles accurately and reducing semantic drift.

#### 4.8.3 Structural and Format Constraints

To further improve the stability of report generation, the outputs are strictly constrained to a standardized format, including “*tumor microenvironment*” which summarizes key TACS types, and “*description*” that provides a semantically complete natural language explanation. This ensures consistent formatting and enhances clinical relevance.

### 4.9 Implementation Details

#### 4.9.1 Virtual Staining

The virtual staining network was trained using an unsupervised cyclic adversarial learning framework between the MPM and H&E image domains. The virtual staining dataset comprised 24 070 image patches (512 × 512 pixels) extracted using a sliding window with 50% overlap after excluding noisy or artifact‐contaminated regions. Because the H&E images were obtained from adjacent tissue sections rather than being pixel‐wise registered to the MPM images, the model was trained in an unpaired image‐to‐image translation setting. The dataset was strictly partitioned at the patient level into a training set (6434 patches) and an independent testing set (17 636 patches), with no patient overlap between the two subsets. The network was trained using Adam (β_1_ =  0.5, β_2_ =  0.999) with a learning rate of 0.0002, maintained for 100 epochs, then linearly decayed to zero over the next 100 epochs. The weight coefficients of the loss terms in Equation (1) were empirically set as follows: λ_*adv*_ =  1, λ_*cycle*_ =  10, λ_*identity*_ =  5, and λ_*SSIM*_ =  1. These values were used in all virtual staining experiments unless otherwise specified. Implementation was based on PyTorch (Python 3.7.16) on an NVIDIA RTX 3090 GPU with CUDA 11.1.

#### 4.9.2 Tumor Margin Visualization

To alleviate the impact of class imbalance, particularly the underrepresentation of the TACS8 category, we applied a stochastic data augmentation pipeline during training. This included geometric transformations (random cropping, rotation, flipping, and scaling) and photometric perturbations (Gaussian noise, blurring, and color jittering). These augmentations were used to increase the diversity of the minority‐class samples and reduce overfitting. This expanded the dataset to 20 000 images, which were finally divided into training, validation, and test sets in a 7:2:1 ratio. The classifier was trained using cross‐entropy loss and AdamW optimizer with the learning rate 1 × 10^−4^ for 100 epochs. We conducted tumor margin diagnosis experiments using PyTorch (Python 3.10.0) with an RTX 3090 GPU (CUDA 11.8).

#### 4.9.3 MPM‐Language Model

Fine‐tuning of Qwen2‐VL‐2B is conducted in the environment comprised of Python 3.12, PyTorch 2.5.1 on Ubuntu 22.04, with training performed on an NVIDIA H20 GPU (96 GB VRAM, CUDA 12.4).

### 4.10 Evaluation Protocols

To evaluate the clinical accuracy and applicability of the proposed method, we conducted a systematic evaluation from two perspectives: margin diagnosis and MPM captioning.

For TME classification and margin visualization, we benchmarked our method against four representative deep learning models: ResNet (CNN‐based) [28], MambaMed (state‐space architecture) [27], and EMO (attention‐free transformer) [29]. These models are recognized for their robust feature extraction and generalization capabilities in medical image analysis. Classification performance was quantified using standard metrics: accuracy, precision, recall, and F1‐score [52]. For margin visualization, spatial prediction quality was assessed using the mean intersection‐over‐union (mIoU) and Dice coefficient [53], which respectively evaluate the overlap and geometric fidelity between predicted boundaries and ground‐truth annotations.

To evaluate the linguistic expressiveness and clinical relevance of MPM image captioning, we compared our approach with four representative LLMs: Gemini 2.5 Pro [54], GPT‐4o [44], Qwen2.5 Max [55], and Llava‐Med (an open‐source model specialized in medical image captioning) [30]. For fairness, all baseline models were provided with the same shared inputs, including the same MPM ROI image, the textual definitions and pathological significance of TACS structures, and the required output format constraints (e.g., structured sections such as TACS summary and microscopic description). In MarginPath, additional probability‐based semantic descriptors and MPM captions were generated by the upstream modules of the proposed framework and used as method‐specific intermediate inputs for final report generation.

To systematically assess the generated reports, evaluations were conducted across two distinct dimensions: linguistic structure and medical content. Linguistic quality was measured using standard natural language generation metrics, including BLEU‐1 to BLEU‐4 [56], ROUGE‐L [57], and Meteor [58], to assess the n‐gram overlap between generated reports and reference descriptions. Clinical and semantic fidelity was evaluated via domain‐specific metrics: semantic similarity, term coverage, term‐level F1 score, and average description length. Collectively, these metrics quantify the completeness of generated content, the appropriateness and coverage of medical terminology, and the controllability of report structure and length.

## Author Contributions

S.W., W.L., and J.C. designed and directed the study; S.W., J.S., X.Z., F.X., and C.L. set up the MarginPath system; X.H., J.P., and X.Z. performed quantitative analysis; S.W. and X.H. performed multiphoton imaging experiments; D.K., X.W., R.L., and F.H. provided specimens; S.W., W.L., J.S., X.H., D.K., and X.Z. wrote the manuscript. A.W., H.C., Q.Z., R.L., and J.C. reviewed and revised the manuscript.

## Conflicts of Interest

The authors declare no conflicts of interest.

## Supporting information




**Supporting File**: advs75709‐sup‐0001‐SuppMat.docx.

## Data Availability

The data that support the findings of this study are available on request from the corresponding author. The data are not publicly available due to privacy or ethical restrictions. The code, pretrained models, and relevant resources of the proposed MarginPath are publicly available at https://github.com/AI‐XPM‐group/MarginPath.
