# Supplementary material for: From Label‐Free Multiphoton Imaging to Pathological Reports: A Vision‐Language Breast Cancer Margin Pathological Diagnosis System
Source: Adv Sci (Weinh). 2026 May 15:e75709. Online ahead of print. doi: 10.1002/advs.75709 (PMC13336036; doi:10.1002/advs.75709)
Supplement: Supplementary file 1 — Supporting File: advs75709‐sup‐0001‐SuppMat.docx. [file ADVS-9999-e75709-s001.docx]

**Supplementary Information**

**From Label-free Multiphoton Imaging to Pathological Reports: A Vision-Language Breast Cancer Margin Pathological Diagnosis System**

Shu Wang^1, 2, 3, #^, Jingze Su^4, #^, Xiahui Han^3, #^, Deyong Kang^5, #^, Xiao Zhang^4^, Fei Xu^1^, Changzu Liu^1^, Junlin Pan^1^, Xingfu Wang^6^, Qiaohui Zhan^7^, Aimin Wang^8^, Feng Huang^1^, Heping Cheng^2^, Wenxi Liu^4, *^, Ruolan Lin^9, *^, Jianxin Chen^3, *^

^1^School of Mechanical Engineering and Automation, Fuzhou University, Fuzhou 350108, China.

^2^National Biomedical Imaging Center, State Key Laboratory of Membrane Biology, Institute of Molecular Medicine, Peking-Tsinghua Center for Life Sciences, College of Future Technology, Peking University, Beijing 100871, China.

^3^Key Laboratory of OptoElectronic Science and Technology for Medicine of Ministry of Education, Fujian Provincial Key Laboratory of Photonics Technology, Fujian Normal University, Fuzhou 350007, China.

^4^College of Computer and Data Science, Fuzhou University, Fuzhou 350108, China.

^5^Department of Pathology, Fujian Medical University Union Hospital, Fuzhou 350001, China.

^6^Department of Pathology, The First Affiliated Hospital of Fujian Medical University, Fuzhou 350005, China.

^7^Department of Breast Surgery, The Second Affiliated Hospital of Xiamen Medical College, Xiamen 361000, China.

^8^State Key Laboratory of Photonics and Communications, School of Electronics, Peking University, Beijing 100871, China.

^9^Department of Radiology, Fujian Medical University Union Hospital, Fuzhou 350001, China.

^#^These authors contributed equally to this work.

^*^Correspondence: Wenxi Liu (wenxiliu@fzu.edu.cn), Ruolan Lin (ruolan.12@foxmail.com), and Jianxin Chen (chenjianxin@fjnu.edu.cn).

# **Table of contents**

**Supplementary Note 1. User study design of MarginPath report.**

**Supplementary Note 2. Clinical reasoning question-answering for breast cancer diagnosis**.

**Supplementary Note 3. Focused observer analysis on heatmap-highlighted peri-margin ROIs.**

**Supplementary Figure 1. Comparison of virtual H&E image across different virtual staining models.**

**Supplementary Figure 2. MPM imaging of breast tissue structures and TACS.**

**Supplementary Figure 3. Quantitative comparison of collagen fiber features among TACS4 to TACS8.**

**Supplementary Figure 4. Margin visualization capabilities of MarignPath.**

**Supplementary Figure 5. A margin pathological report for a representative luminal B breast cancer case generated by MarginPath.**

**Supplementary Figure 6. Comparative analysis of model-generated descriptions for the tumor-stroma boundary in MPM images.**

**Supplementary Figure 7. Breast cancer question-answering capabilities of MarginPath.**

**Supplementary Figure 8. Example of MarginPath's Q&A system interpreting TME results for a patient.**

**Supplementary Figure 9. Example of MarginPath's Q&A system for building a TACS-based prognostic model.**

**Supplementary Figure 10. Quantitative evaluation of heatmap-guided ROI selection in diagnostically challenging peri-margin cases.**

**Supplementary Figure 11. Schematic illustration of the MarginPath virtual staining network.**

**Supplementary Figure 12 Flowchart of the interactive ROI selection.**

**Supplementary Figure 13. Architecture and training strategy of the MPM-Language model.**

**Supplementary Figure 14. Word frequency and length distribution of MPM description generation dataset.**

**Supplementary Table 1. Quantitative comparison of text fluency and form in pathological report generation across models.**

**Supplementary Table 2. Quantitative comparison of information coverage and accuracy in pathological report generation across models.**

**Supplementary Table 3. The details of MPM-to-H&E virtual staining dataset.**

**Supplementary Table 4. The details of Tumor margin visualization dataset.**

**Supplementary Table 5. The details of MPM description generation dataset.**

**Supplementary Table 6. The rules of mapping the classification probability of TACS to qualitative description.**

**Supplementary Table 7. The rules of mapping the classification probability of tumor to qualitative description.**

## **Supplementary Note 1 User study design of MarginPath report.**

**1. Study rationale and participant recruitment**

Subjective evaluation is an effective method for assessing the quality of biomedical image analysis outputs, as it captures expert perceptual judgment of visual and textual information. To comprehensively evaluate our proposed MarginPath system, we conducted an anonymous online survey with nine participants from three relevant backgrounds: pathology, computer vision, and biophotonics.

**2. Evaluation framework**

All participants completed three sequential evaluation tasks, providing scores on a scale of 0 to 10 for each criterion. The specific tasks and scoring criteria were as follows:

**(1) Virtual H&E staining image quality:**

Participants evaluated the overall visual fidelity of MarginPath-generated virtual high-resolution H&E images compared to paired real H&E stains from six independent cases.

(a) 0-2 points (Unacceptable): Tissue structures (e.g., cells) are uninterpretable, severely hindering observation.

(b) 3-4 points (Major Revisions Needed): Major stylistic discrepancies, with large areas of under-/abnormal staining that hinder observation.

(c) 5-6 points (Marginally Acceptable): Minor under-/abnormal staining in some areas, slightly hindering observation.

(d) 7-8 points (Good): Slight stylistic differences that do not hinder observation.

(e) 9-10 points (Excellent): Virtually indistinguishable from real staining; no abnormal regions.

**(2) Margin localization accuracy**

Participants assessed the precision of tumor margin positioning in heatmap visualizations across 19 MPM ROI-based classifications.

(a) 0-2 points (Unacceptable): Major deviations; provides no reliable basis for clinical decisions.

(b) 3-4 points (Major Revisions Needed): Obvious errors and inaccuracies in key areas, requiring major re-evaluation.

(c) 5-6 points (Marginally Acceptable): Generally usable but with local inaccuracies that slightly interfere with clinical judgment.

(d) 7-8 points (Good): Accurate localization with clear representation of key areas and minimal errors, effectively supporting judgment.

(e) 9-10 points (Excellent): Precise, error-free localization with complete detail, reliably guiding critical clinical decisions.

**(3) Image description report quality**

Participants analyzed 19 textual reports generated from MPM ROIs, focusing on linguistic fluency (coherence, terminology) and the accuracy of TME descriptions (key pathological features).

(a) 0-2 points (Unacceptable): Seriously flawed. Missing/contradictory margin descriptions, fragmented/colloquial language, and ambiguous conclusions provide no clinical utility; may be misleading.

(b) 3-4 points (Major Revisions Needed): Provides only minimal information. Imprecise descriptions, awkward organization with improper terms, and overly generalized conclusions require substantial revision.

(c) 5-6 points (Marginally Acceptable): Correct core diagnosis but with significant information gaps, non-standard descriptions, or insufficient precision, creating clinical uncertainty.

(d) 7-8 points (Good): Meets basic accuracy requirements. Correct margin status determination and professional terminology are used, but writing is formulaic and lacks critical detail or added insight.

(e) 9-10 points (Excellent): Achieves professional standards. Accurate margin descriptions, fluent and logical language, and precise diagnoses that include clinically significant details to reliably guide decision-making.

**3. Evaluation framework and results**

Participants were allowed to review and modify their scores before final submission. Overall, the quantitative ratings (mean±SEM, scale 0-10) from the nine evaluators demonstrated strong performance across all three key metrics. Virtual H&E staining image quality scored 8.09±0.68, margin localization accuracy scored 9.04±1.10, and image description report quality scored 9.01±1.06. These results demonstrate that the MarginPath system can generate virtual H&E images of good quality, achieve accurate margin localization, and provide professional, clear, and fluent diagnostic image description reports.

## **Supplementary Note 2 Clinical reasoning question-answering for breast cancer diagnosis.**

To meet the personalized consultation needs of postoperative patients, we propose and implement an LLM-based question-answering model to further enhance the proposed MarginPath. The core development pipeline is as follows: First, a high-quality dataset rich in clinical reasoning is constructed. Subsequently, this dataset is utilized to fine-tune the model using reinforcement learning algorithm. During this process, a multi-metric reward function is designed to guide the model's optimization.

**Dataset construction**: We first extract breast disease-related question-answer pairs from the MedQA-USMLE^[1]^ and MedMCQA^[2]^ datasets. We select 1,510 high-quality breast cancer-related Q&A pairs for use. Sourced from medical licensing exams, these resources ensure strong clinical relevance.

**Reasoning chain generation**: We use an LLM to generate chain-of-thought (CoT) reasoning for each question. In practice, we employ DeepSeek-R1^[3]^ for this task. Samples with correct final answers retain their reasoning paths. These paths simulate clinical diagnostic thinking, covering context understanding, pathophysiology, differential diagnosis, and guideline-based decisions. All reasoning texts are manually verified for accuracy and coherence.

**Model fine-tuning with GRPO**: We fine-tune the Qwen2.5-3B model^[4]^ using the group relative policy optimization (GRPO) algorithm^[5]^ on our reasoning-enhanced dataset. GRPO is a recently proposed policy optimization algorithm designed to guide LLMs toward producing high-quality responses aligned with human preferences and task objectives. It employs a relative advantage evaluation mechanism, which significantly improves reasoning performance in complex language tasks.

**Multi-metric reward function**: To precisely guide the model's optimization during the reinforcement learning stage, we employ a composite reward function. This function aims to ensure that the model's responses are not only medically accurate but also meet the high standards for clinical utility in terms of logical reasoning, structural organization, and linguistic expression. Its core mechanism involves generating a comprehensive reward value through the weighted fusion of signals from four independent evaluation dimensions. This integrated reward value then serves as the objective for optimization.

The concrete reward calculation is shown below:

|  | $R_{total}= w_{sem}\times R_{sem}+ w_{flu}\times R_{flu}+w_{str}\times R_{str}+w_{dep}\times R_{dep},$ | (1) |
| --- | --- | --- |

The details of each component metric are described below:

Semantic Correctness ($R_{sem}$): This metric assesses the factual consistency between generated and reference answers using the STSB-RoBERTa-large cross-encoder,^[6]^ which outputs a similarity score between 0 and 1. Empty responses are assigned a default score of -1 to enforce factual accuracy. The weight for this semantic score ($w_{sem}$) is set to 0.4.

Linguistic Fluency ($R_{flu}$): By leveraging BioGPT^[7]^ to calculate perplexity, this metric promotes fluent and natural text generation. The raw perplexity scores are then normalized to a value between 0 and 1 to produce the final reward, with the associated weight ($w_{flu}$) set to 0.3.

Structural Completeness ($R_{str}$): This metric enforces a consistent output structure by verifying the presence of distinct "reasoning" and "answer" components, assigning a score of 1.0 for both, 0.5 for one, and 0.0 for neither. The weight for this structural reward ($w_{str}$) is set to 0.1.

Reasoning Depth ($R_{dep}$): To ensure the model produces sufficiently developed reasoning, this metric uses length as a proxy for comprehensiveness and interpretability, rewarding a score of 1.0 for reasoning components exceeding 1,000 characters with a weight ($w_{dep}$) of 0.2.

In each policy update step, the final $R_{total}$ values corresponding to the batch of generated responses are standardized and subjected to boundary clipping, strictly constraining them to the [-1.0, 1.0] interval. This design ensures the model receives stable and discriminative gradient signals during training, effectively optimizing it towards the multi-objective goal of generating responses that are "accurate, fluent, well-structured, and deeply reasoned."

**Optimization outcomes**: As shown in Extended Data Figure 3c, reinforcement learning with this reward function leads to a substantial improvement in the LLM's accuracy on specialized breast cancer question-answering tasks.

## **Supplementary Note 3 Focused observer analysis on heatmap-highlighted peri-margin ROIs.**

**1. Study rationale**

Subtle tumor foci in peri-margin regions are difficult to identify during rapid intraoperative assessment, particularly when scattered tumor cells are embedded within complex stromal backgrounds. To evaluate whether the MarginPath heatmap can highlight diagnostically relevant high-risk regions, we performed a focused observer analysis on heatmap-highlighted peri-margin ROIs.

**2. ROI selection and reference standard**

For this analysis, suspicious tumor-boundary/peri-margin regions were first identified on the MPM heatmap, especially within the yellow pseudo-colored transition zones indicating potentially high-risk areas. The selected ROIs were then mapped to the corresponding real H&E-stained images. Pathologists performed blinded assessment on these matched real H&E images to determine the presence or absence of tumor cells. The reference standard was defined according to the expert consensus on the matched H&E images.

**3. Quantitative evaluation and results**

We selected 26 challenging peri-margin cases from the test set. The diagnostic relevance of the heatmap-highlighted ROIs was evaluated by comparing the model-predicted probabilities with the reference labels derived from the matched true H&E images. As shown in the Receiver Operating Characteristic (ROC) curve (Supplementary Figure. 10b), MarginPath demonstrates strong discriminative performance with an overall AUC of 0.940. On this subset, MarginPath achieves an overall accuracy of 92.31%, a sensitivity of 91.67%, and a specificity of 92.86% at the optimal probability threshold of 0.591. These findings support the potential value of the heatmap as an attention-guiding tool for localizing subtle tumor-positive peri-margin regions.


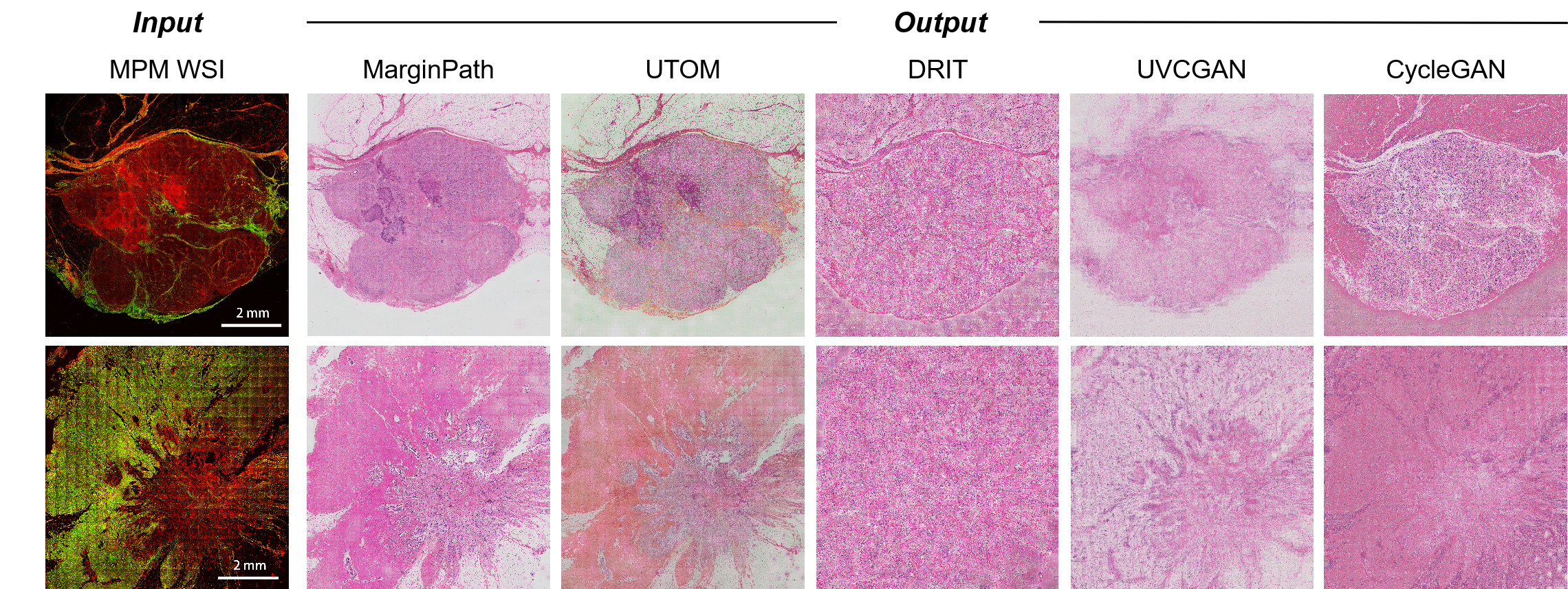


## **Supplementary Figure 1 Comparison of virtual H&E image across different virtual staining models.** The output of the proposed MarginPath model demonstrates superior performance in restoring fine tissue structures (e.g., tumor morphology and stromal texture) and staining styles compared to other competing methods.

**
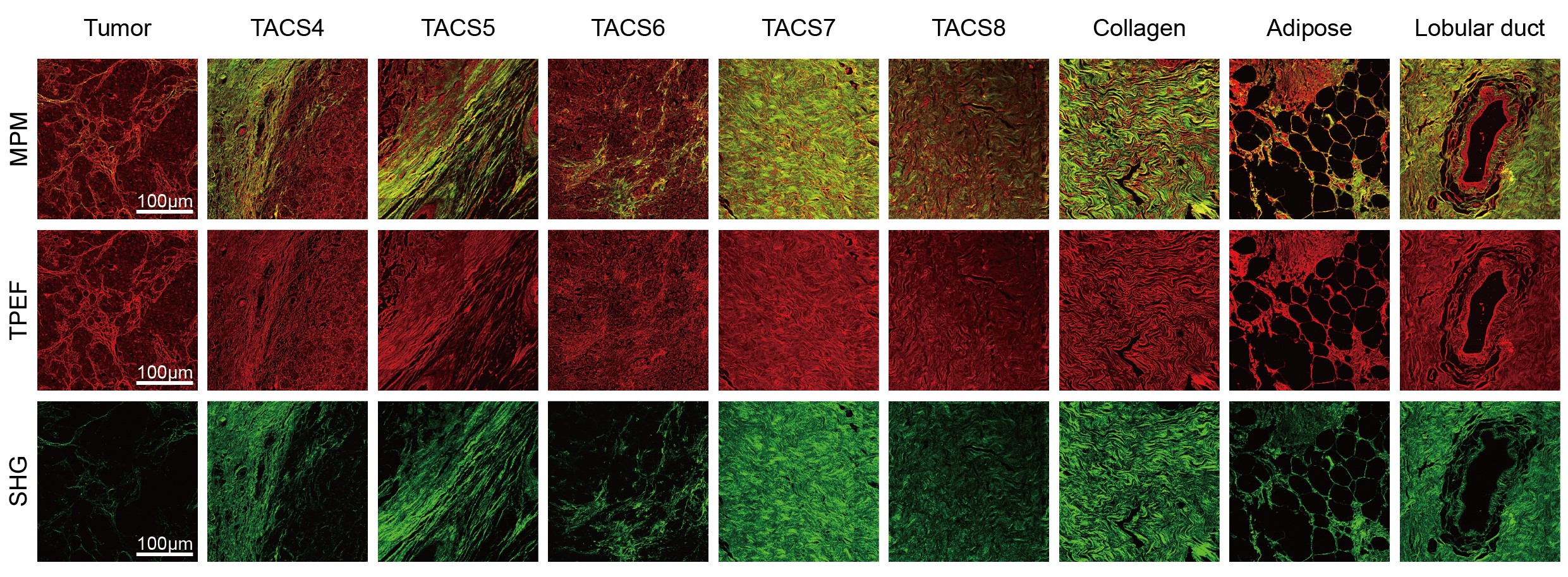
**

## **Supplementary Figure 2 MPM imaging of breast tissue structures and TACS.** This figure presents representative MPM, two-photon excited fluorescence (TPEF), and second harmonic generation (SHG) images, showcasing normal breast structures (lobular ducts, collagen, adipose tissue) alongside tumor tissue and TACS-4 to TACS-8. The side-by-side comparison highlights distinct morphological features provided by each modality, serving as a direct visual reference for characterizing the breast tissue microenvironment and the specific optical signatures of progressive collagen remodeling in breast cancer.


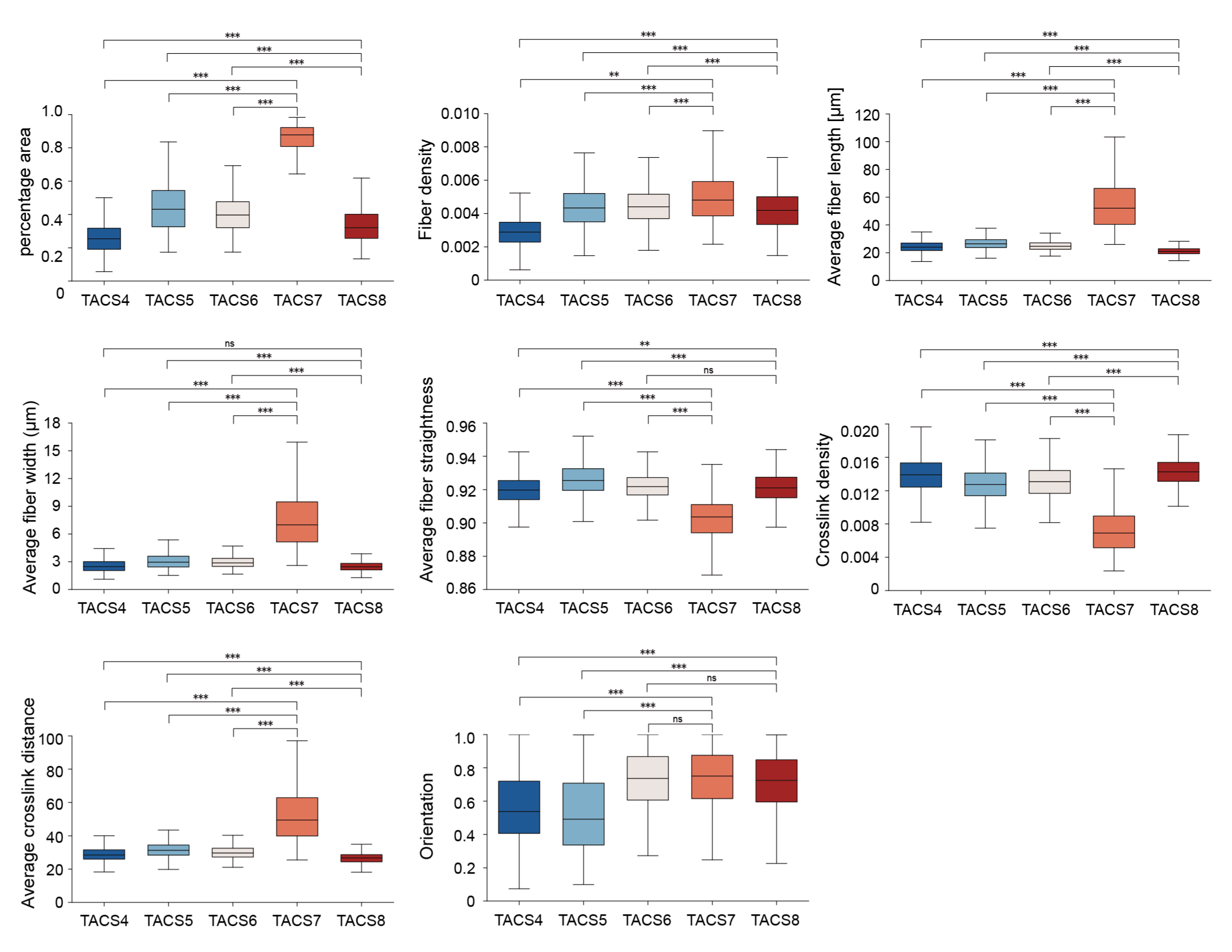


## **Supplementary Figure 3** **Quantitative comparison of collagen fiber features among TACS4 to TACS8.** Boxplots depict distributions of eight parameters: percentage area, fiber density, average fiber length, average fiber width, average fiber straightness, crosslink density, average crosslink distance, and orientation. Statistical significance between groups was assessed, with ns indicating no significance, ^*^<0.05, ^**^*p*<0.01, and ^***^*p*<0.001. These results highlight distinct quantitative characteristics across different TACS patterns. *n*=16,654 patches.


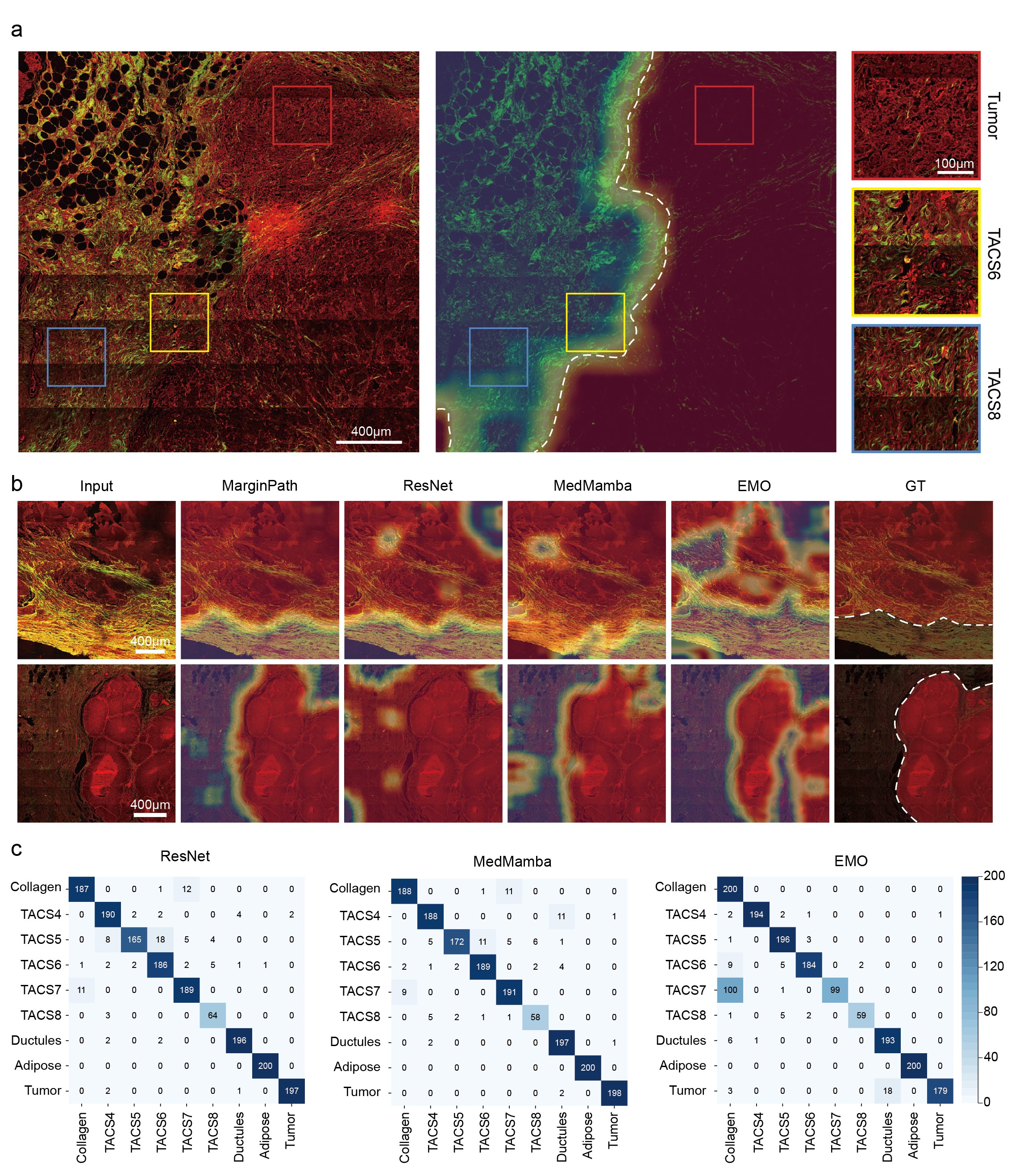


## **Supplementary Figure 4** **Margin visualization capabilities of MarignPath.** a Large-scale margin prediction heatmap of an invasive breast cancer specimen. Red box: tumor in high-probability region. Yellow box: TACS4 in moderate-probability region. Blue box: TACS8 in low-probability region. b Margin visualization results from different methods on two representative ROIs. c TME features classification performance of ResNet, MedMamba, and EMO. While MedMamba and ResNet frequently confuse morphologically similar classes, EMO exhibits confusion between TACS7 and normal collagen, and misclassifies lobular ducts as tumor tissue.

**
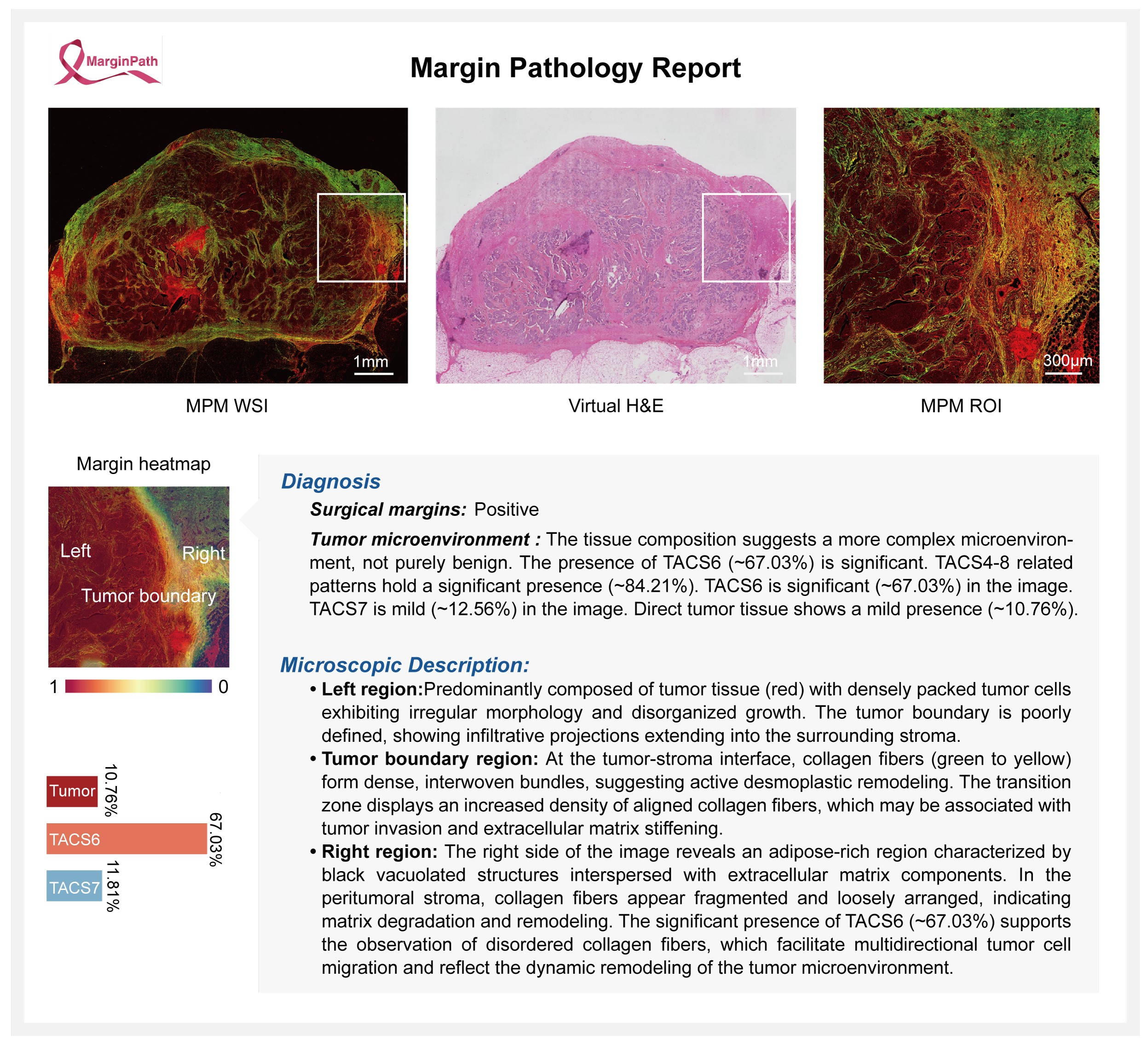
**

## **Supplementary Figure 5 A margin pathological report for a representative luminal B breast cancer case generated by MarginPath.**

**
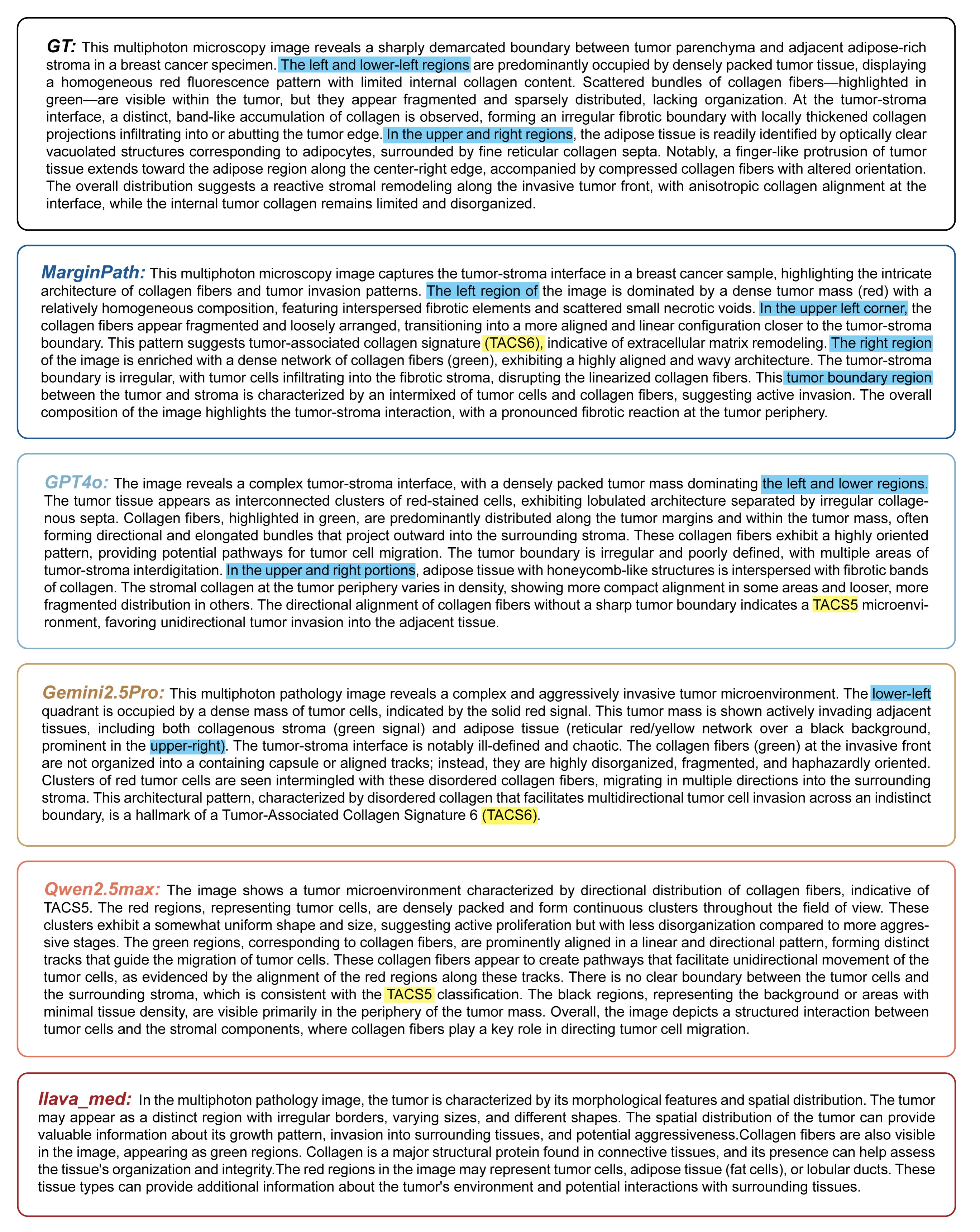
**

## **Supplementary Figure 6 Comparative analysis of model-generated descriptions for the tumor-stroma boundary in MPM images.** To evaluate descriptive accuracy, model outputs were compared against a detailed ground truth (GT) description, with blue and yellow annotations highlighting location and TACS information, respectively. MarginPath demonstrates superior precision by accurately capturing core GT features, including "collagen architecture at the tumor-stroma interface" and "tumor infiltration," and is the only model to correctly identify the TACS6 invasion pattern, aligning with the GT's biological interpretation. In contrast, GPT-4o and Qwen2.5-Max misclassify the pattern as TACS-5, while Gemini-2.5-Pro's identification of TACS-6 is inconsistent with its description of collagen as "highly disorganized", contradicting the GT's report of anisotropic alignment. LLaVA-Med provides only a generalized overview, lacking critical microenvironmental details. This comparison indicates that MarginPath holds a distinct advantage in translating MPM image features into accurate pathological concepts, delivering more structurally precise locational descriptions and more reliable TACS information.


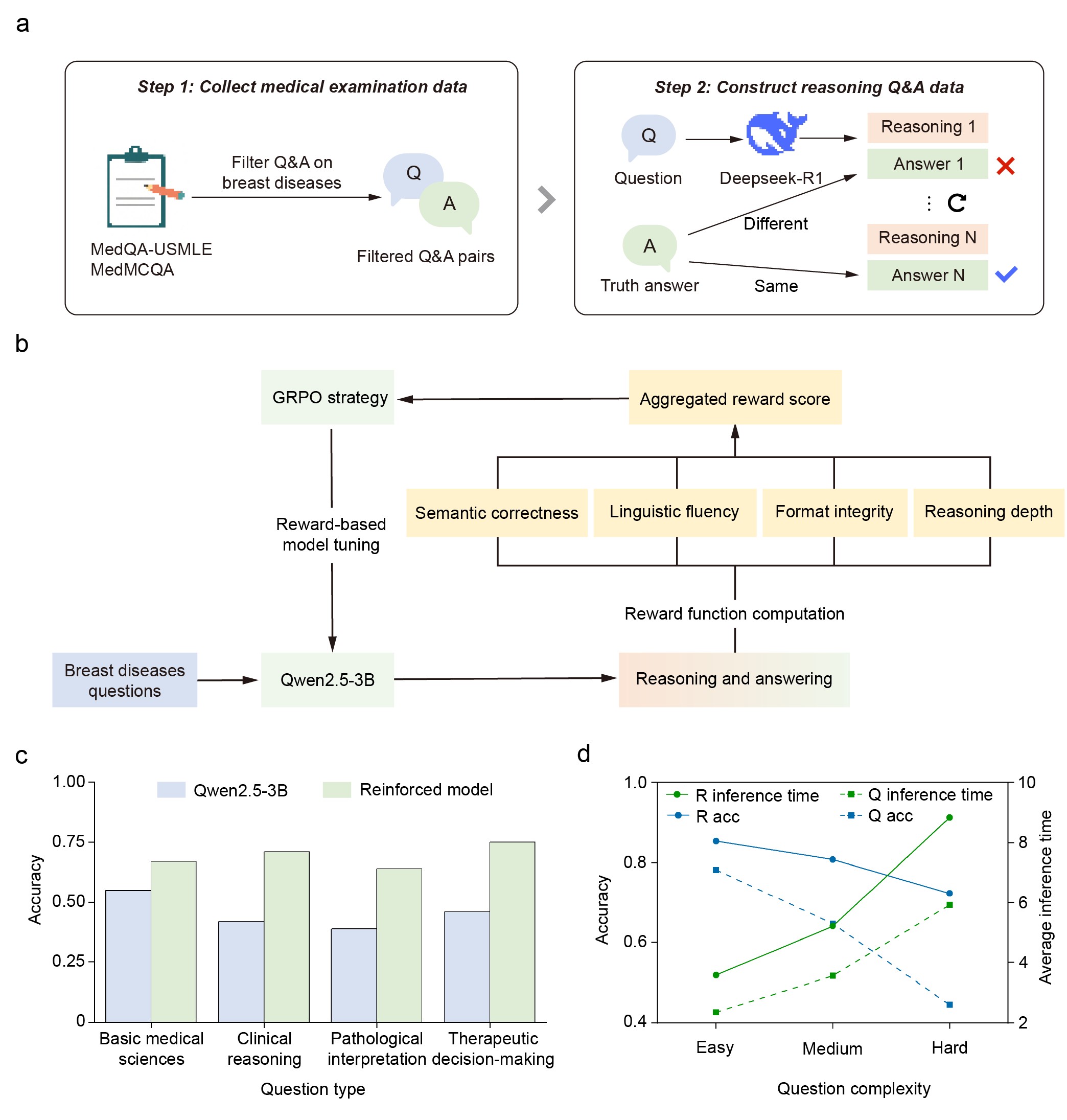


## **Supplementary Figure 7** **Breast cancer question-answering capabilities of MarginPath.** a Pipeline for constructing the breast cancer reasoning Q&A dataset. Real clinician-curated medical exam questions were collected, followed by rationale-inclusive dataset generation using DeepSeek-R1. b Fine-tuning workflow of Qwen2.5-3B with GRPO to enhance reasoning and response generation. c Accuracy comparison of fine-tuned versus baseline Qwen2.5-3B models across breast pathology question types. d Performance gains of the reinforced model over the base model by question complexity tier, with corresponding inference latency increases.


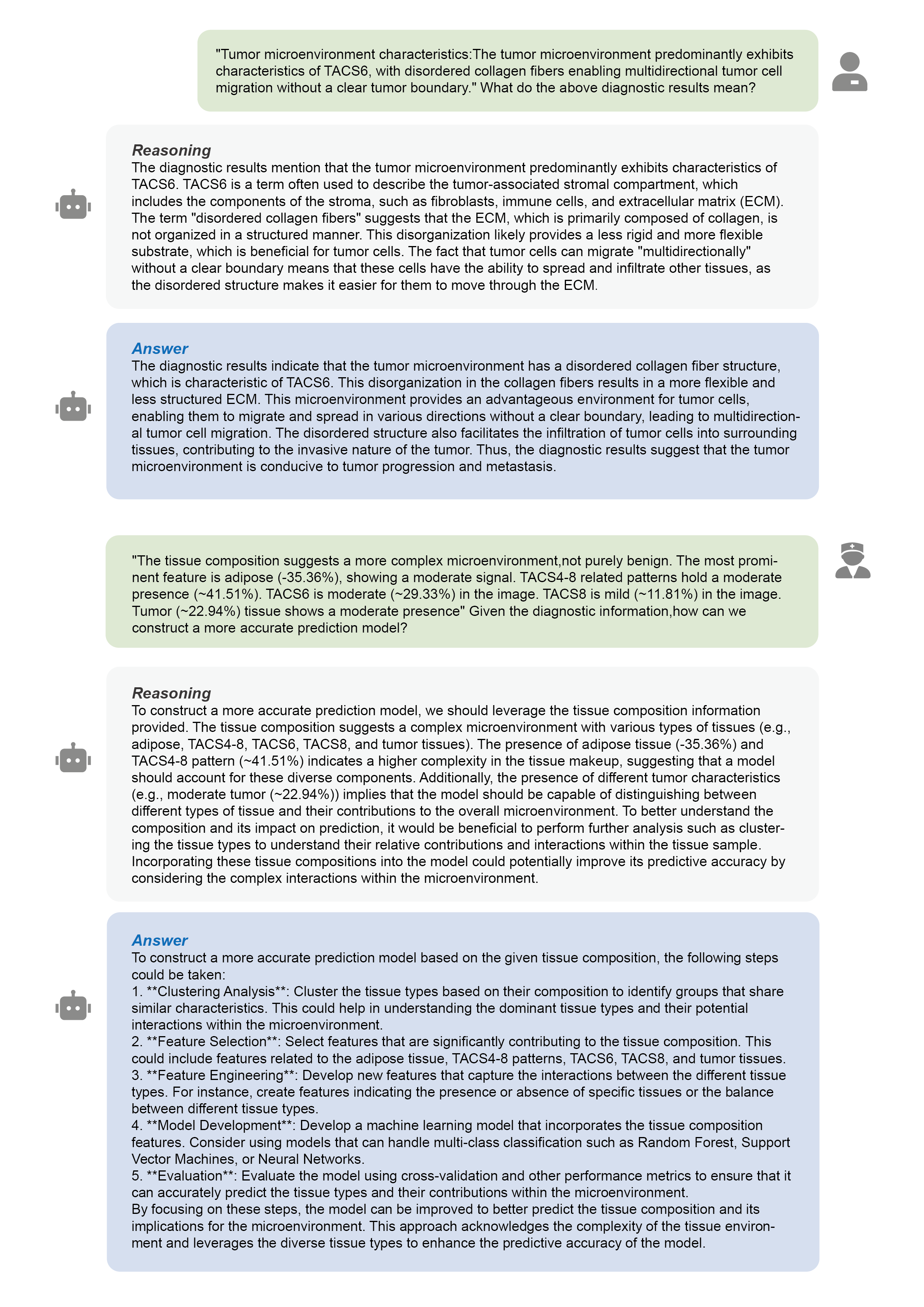


## **Supplementary Figure 8 Example of MarginPath's Q&A system interpreting TME results for a patient.** This example presents MarginPath's complete response to a patient's inquiry regarding TME-based diagnostics. The response is divided into two parts: Part 1 (Reasoning), which explains relevant medical concepts (e.g., TACS6, disordered collagen); and Part 2 (Answer), which clearly summarizes the diagnostic implications, specifically how these TME features may promote tumor invasion and metastasis.


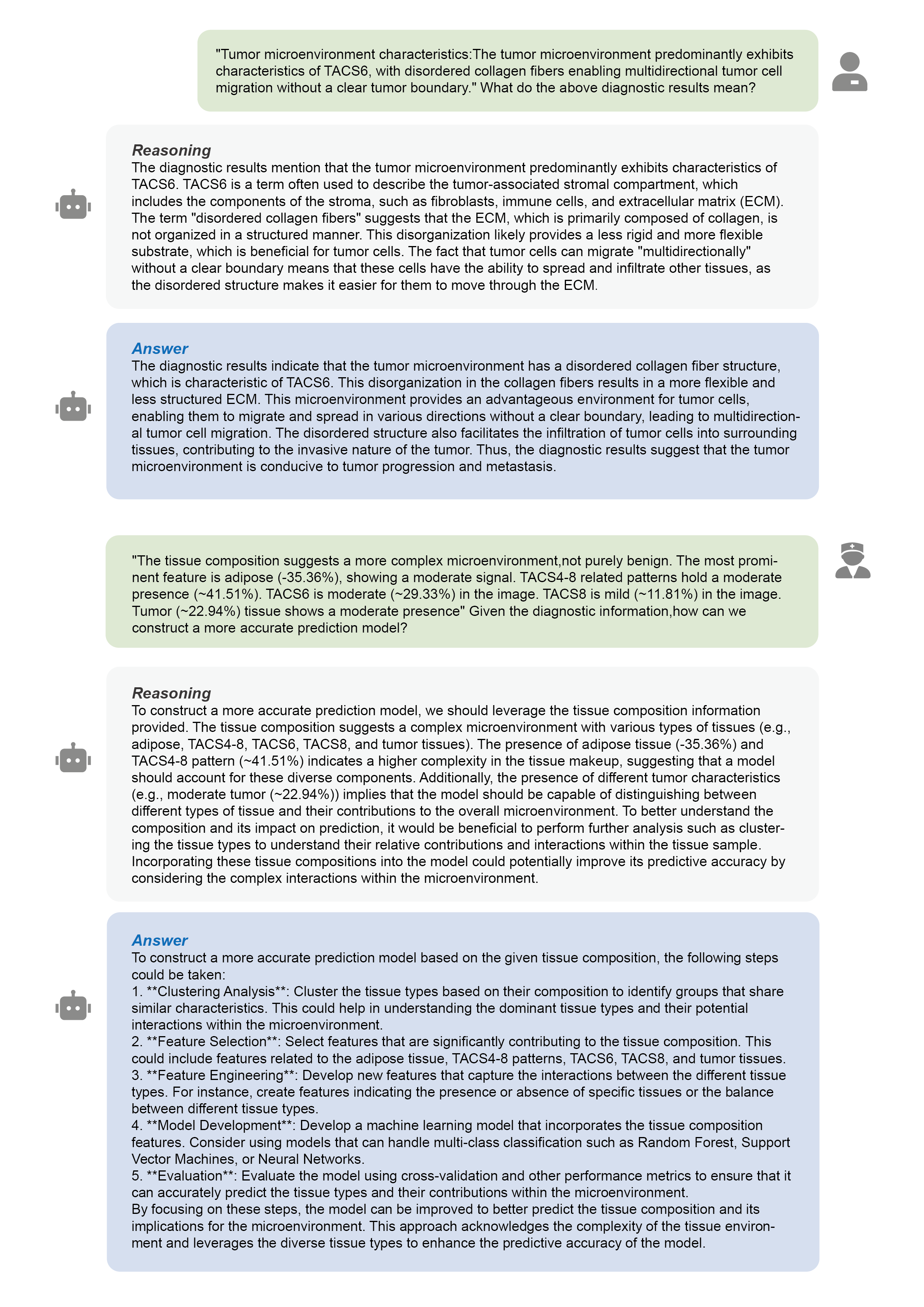


## **Supplementary Figure 9 Example of MarginPath's Q&A system for building a TACS-based prognostic model for a clinician.** This example presents the system’s response to a clinician’s inquiry about developing a prognostic model. The response is divided into two parts: Part 1 (Reasoning), which analyzes the patient’s TME components and the key elements required for model development; and Part 2 (Answer), which outlines a five-step modeling framework comprising clustering analysis, feature selection, feature engineering, model development, and evaluation.


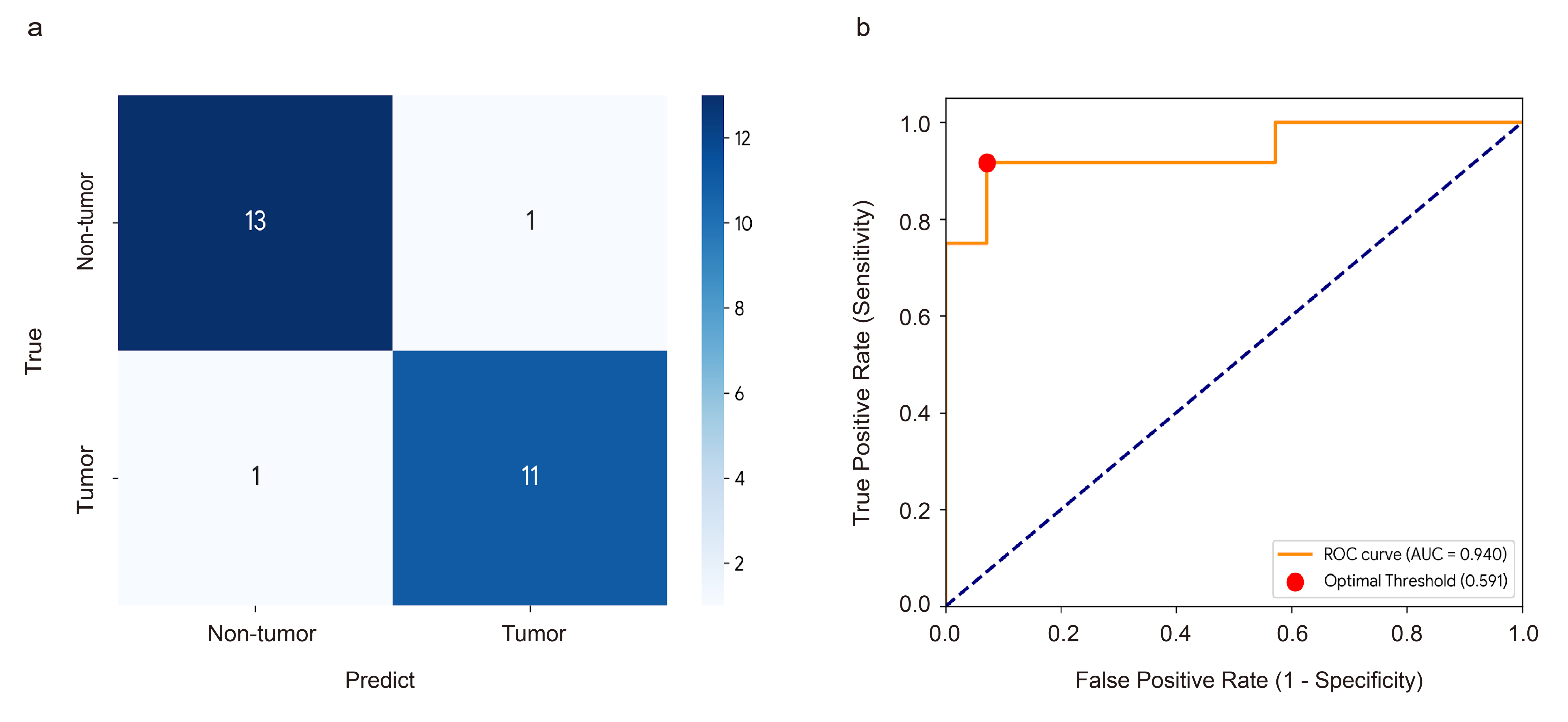


## **Supplementary Figure 10 Quantitative evaluation of heatmap-guided ROI selection in diagnostically challenging peri-margin cases.** (a) Confusion matrix of the 26 selected challenging ROIs at the optimal probability threshold of 0.591, demonstrating high sensitivity (91.67%) and specificity (92.86%) for localizing subtle tumor foci. (b) Receiver operating characteristic (ROC) curve for margin prediction in these difficult cases, achieving an Area under the curve (AUC) of 0.940.


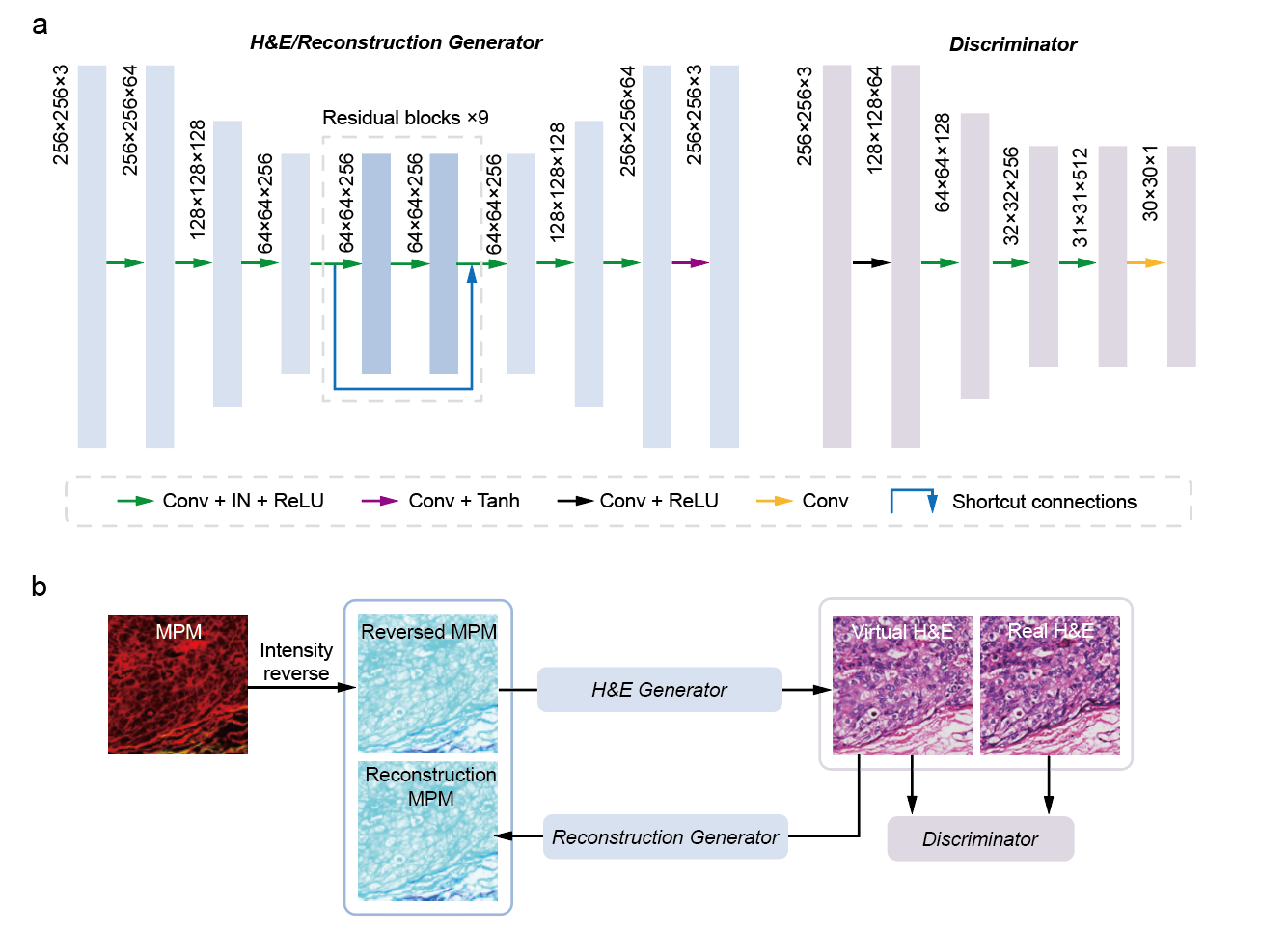


## **Supplementary Figure 11 Schematic illustration of the MarginPath virtual staining network. a.** The H&E generator is composed of downsampling layers, residual blocks, and upsampling layers, while the discriminator consists of 6 convolutional layers. Each rectangle represents a multi-channel feature map, with its spatial dimensions and channel number labeled on the left. **b.** The image data flow during the training phase of the virtual staining network.


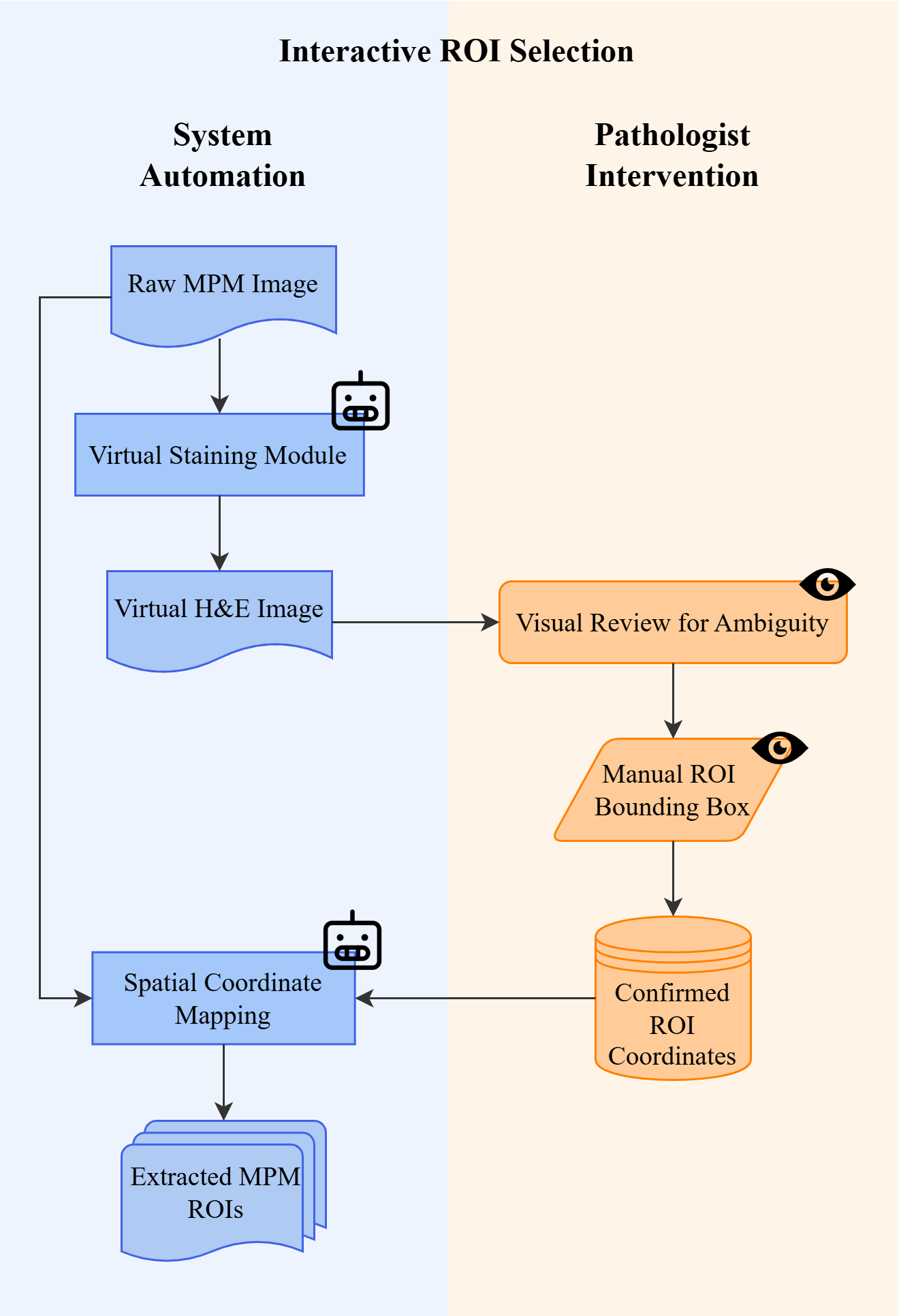


## **Supplementary Figure 12 Flowchart of the interactive ROI selection workflow.** The system adopts a human-in-the-loop paradigm in which a pathologist selects ROIs by identifying diagnostically uncertain regions on virtual H&E images. The system then automatically maps the selected coordinates to the corresponding MPM images and extracts the matched sub-images for subsequent tumor margin visualization.


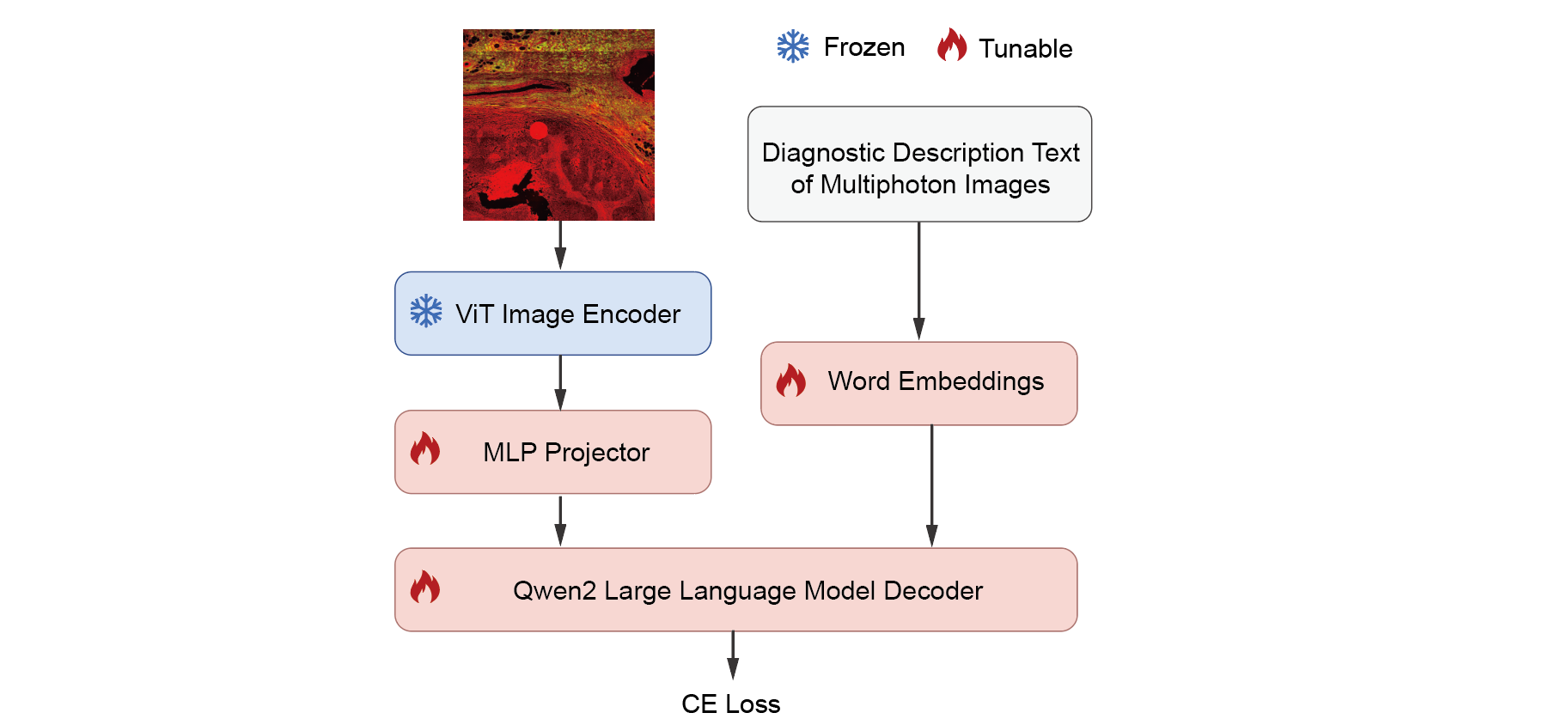


## **Supplementary Figure 13 Architecture and training strategy of the MPM-Language model.** This model is adapted from Qwen2-VL-2B to generate descriptive text for MPM images. Its architecture consists of a ViT image encoder, word embeddings, an MLP projector, and a Qwen2 LLM decoder. During fine-tuning, the image encoder was kept frozen while the remaining components were trained.


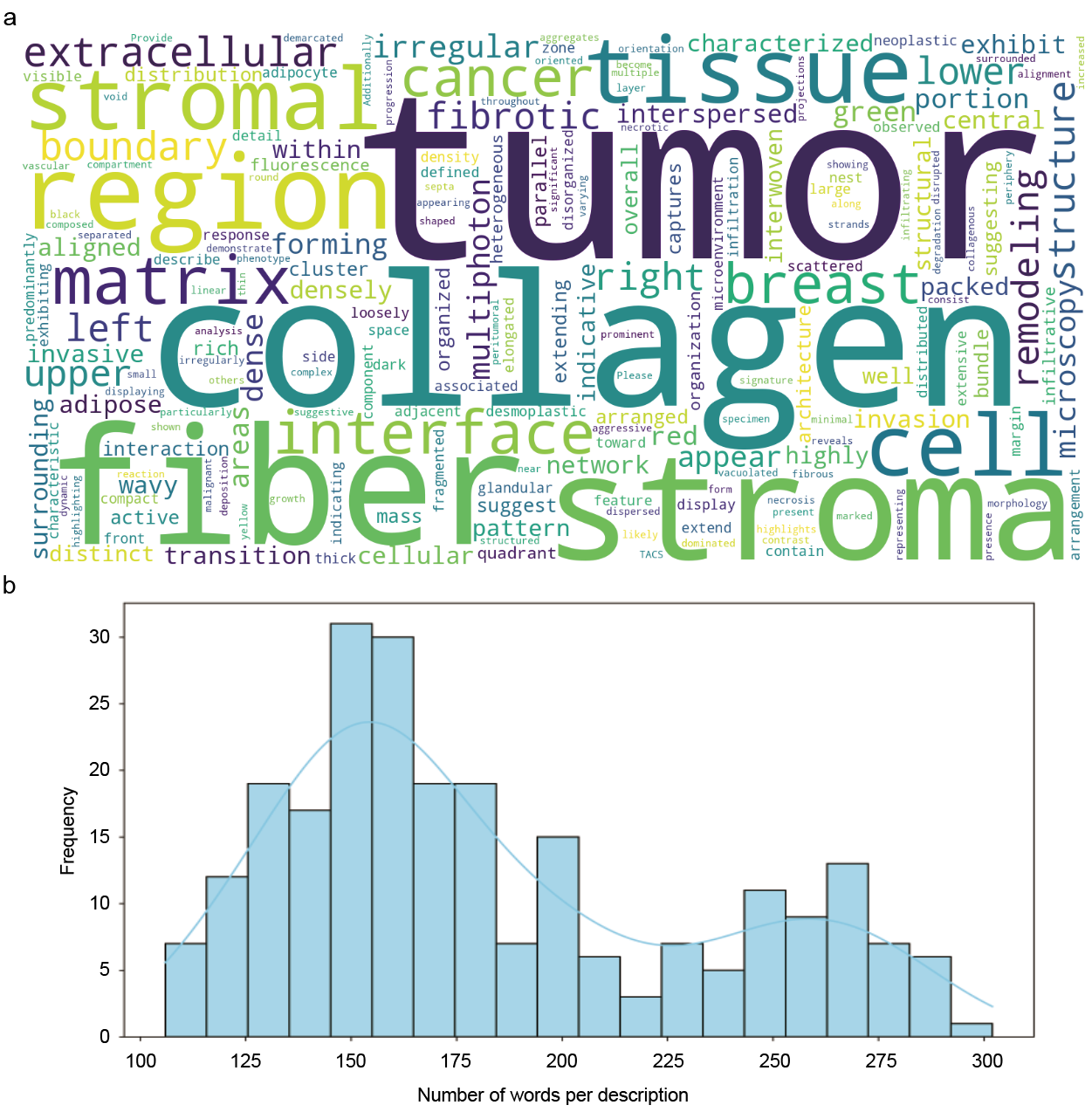


## **Supplementary Figure 14 Word frequency and length distribution of MPM description generation dataset. a.** The word cloud depicts word frequency in the multiphoton image caption corpus, where size and color prominence correspond to occurrence rates. **b.** The histogram reveals a slightly left-skewed distribution of description lengths, which are predominantly concentrated between 120 and 180 words. This concentration indicates strong linguistic consistency and structural regularity across the dataset. A small number of descriptions exceeding 250 words form a long tail on the right, corresponding to more detailed and complex texts.

## **Supplementary Table 1. Quantitative comparison of text fluency and form in pathological report generation across models.**

|  | **BLEU_1** | **BLEU_2** | **BLEU_3** | **BLEU_4** | **ROUGE-L** | **Meteor** |
| --- | --- | --- | --- | --- | --- | --- |
| Gemini2.5Pro | 0.392 | 0.203 | 0.104 | 0.052 | 0.274 | 0.263 |
| GPT-4o | 0.457 | 0.23 | 0.11 | 0.052 | 0.293 | 0.298 |
| Qwen2.5Max | 0.373 | 0.174 | 0.075 | 0.038 | 0.231 | 0.247 |
| Llava-Med | 0.221 | 0.094 | 0.031 | 0.01 | 0.174 | 0.163 |
| **MarginPath** | **0.458** | **0.257** | **0.148** | **0.081** | **0.332** | **0.348** |

## **Supplementary Table 2. Quantitative comparison of information coverage and accuracy in pathological report generation across models.**

|  | **CosSim** | **Term-Recall** | **Term-F1** | **Length Ratio** |
| --- | --- | --- | --- | --- |
| Gemini2.5Pro | 0.791 | 0.447 | 0.569 | 0.826 |
| GPT-4o | 0.831 | 0.506 | 0.605 | 0.898 |
| Qwen2.5Max | 0.744 | 0.282 | 0.412 | 0.592 |
| Llava-Med | 0.679 | 0.31 | 0.439 | 0.863 |
| **MarginPath** | **0.852** | **0.76** | **0.714** | **1.095** |

## **Supplementary Table 3.** **The details of MPM-to-H&E virtual staining dataset.**

| **Patch Number** | **Category** | **Resolution [pixel]** |
| --- | --- | --- |
| 24070 | MPM-H&E Pairs | 512×512 |

## **Supplementary Table 4. The details of Tumor margin visualization dataset.**

| **Patch Number** | **Category** | **Resolution [pixel]** |
| --- | --- | --- |
| 2000 | TACS4 | 512×512 |
| 2000 | TACS5 |  |
| 2000 | TACS6 |  |
| 2000 | TACS7 |  |
| 654 | TACS8 |  |
| 2000 | Lobular Duct |  |
| 2000 | Collagen |  |
| 2000 | Adipose |  |
| 2000 | Tumor |  |

## **Supplementary Table 5. The details of MPM description generation dataset.**

| **ROI Number** | **Category** | **Resolution [pixel]** |
| --- | --- | --- |
| 448 | MPM-text Pairs | 4296×4322 |

## **Supplementary Table 6. The rules of mapping the classification probability of TACS to qualitative description.** Note that, as the significant level is trace, no textual description is required.

| **Probability** | (0,0.05] | (0.05,0.2] | (0.2,0.5] | (0.5,1) |
| --- | --- | --- | --- | --- |
| **Clinical Significance Level (CSL)** | trace | mild | moderate | significant |
| **Description** | N/A | "mildly" | "moderately" | "significantly" |

## **Supplementary Table 7. The rules of mapping the classification probability of tumor to qualitative description.**

| **Probability** | (0,0.05] | (0.05,0.2] | (0.2,1) |
| --- | --- | --- | --- |
| **Clinical Significance Level (CSL)** | mild | moderate | significant |
| **Description** | "mildly" | "moderately" | "significantly" |

# **Reference**

1. Jin, D., Pan, E., Oufattole, N., Weng, W.-H., Fang, H. & Szolovits, P. "What Disease Does This Patient Have? A Large-Scale Open Domain Question Answering Dataset from Medical Exams". *Applied Sciences* **11**, (2021).

2. Pal, A., Umapathi, L. K. & Sankarasubbu, M. "Medmcqa: A large-scale multi-subject multi-choice dataset for medical domain question answering". *Conference on Health, Inference, and Learning* 248-260 (2022).

3. Guo, D., Yang, D., Zhang, H., et al. "Deepseek-r1: Incentivizing reasoning capability in llms via reinforcement learning". *arXiv preprint arXiv:12948*, (2025).

4. Team, Q. "Qwen2 technical report". *arXiv preprint arXiv:10671*, (2024).

5. Shao, Z., Wang, P., Zhu, Q., et al. "Deepseekmath: Pushing the limits of mathematical reasoning in open language models". *arXiv preprint arXiv:03300*, (2024).

6. Reimers, N. & Gurevych, I. "Sentence-BERT: Sentence Embeddings using Siamese BERT-Networks". *Proceedings of the 2019 Conference on Empirical Methods in Natural Language Processing and the 9th International Joint Conference on Natural Language Processing (EMNLP-IJCNLP)* 3982-3992 (2019).

7. Luo, R., Sun, L., Xia, Y., et al. "BioGPT: generative pre-trained transformer for biomedical text generation and mining". *Briefings in Bioinformatics* **23**, bbac409 (2022).
